# Innovative Delivery Systems Loaded with Plant Bioactive Ingredients: Formulation Approaches and Applications

**DOI:** 10.3390/plants10061238

**Published:** 2021-06-18

**Authors:** Anastasia Kyriakoudi, Eleni Spanidi, Ioannis Mourtzinos, Konstantinos Gardikis

**Affiliations:** 1 Laboratory of Food Chemistry and Biochemistry, Department of Food Science and Technology, Faculty of Agriculture, Aristotle University of Thessaloniki, 54124 Thessaloniki, Greece; ankyria@chem.auth.gr (A.K.); mourtzinos@agro.auth.gr (I.M.); 2 APIVITA SA, Industrial Park, Markopoulo, 19003 Athens, Greece; spanidi-e@apivita.com

**Keywords:** delivery systems, encapsulation, bioactive compounds, carriers, extracts, essential oils

## Abstract

Plants constitute a rich source of diverse classes of valuable phytochemicals (e.g., phenolic acids, flavonoids, carotenoids, alkaloids) with proven biological activity (e.g., antioxidant, anti-inflammatory, antimicrobial, etc.). However, factors such as low stability, poor solubility and bioavailability limit their food, cosmetics and pharmaceutical applications. In this regard, a wide range of delivery systems have been developed to increase the stability of plant-derived bioactive compounds upon processing, storage or under gastrointestinal digestion conditions, to enhance their solubility, to mask undesirable flavors as well as to efficiently deliver them to the target tissues where they can exert their biological activity and promote human health. In the present review, the latest advances regarding the design of innovative delivery systems for pure plant bioactive compounds, extracts or essential oils, in order to overcome the above-mentioned challenges, are presented. Moreover, a broad spectrum of applications along with future trends are critically discussed.

## 1. Introduction

**Figure 1:**
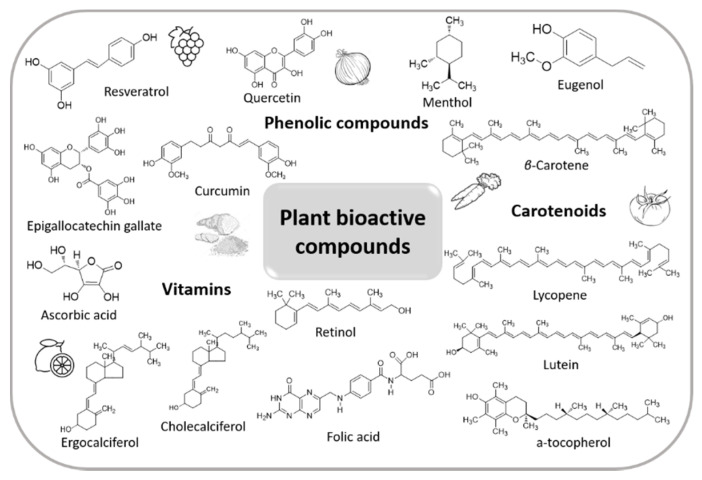
Representative classes of plant-derived bioactive compounds.

Plants produce a wide range of phytochemicals such as phenolic compounds (e.g., phenolic acids, flavonoids, stilbenes, tannins), terpenes (e.g., carotenoids) as well as nitrogen- (e.g., alkaloids) and sulfur-containing compounds (Figure 1). Various biological actions, including antioxidant, anti-inflammatory, anticancer, anti-atherosclerotic, antimicrobial, etc., have been assigned to plant-derived compounds. The latter ones, in the form of either isolated molecules, extracts or essential oils, find numerous applications in food, pharmaceutical and cosmetic industries as natural colorants, flavoring agents, antioxidants, antimicrobials, nutraceuticals, etc. [1]. However, their direct incorporation into various products presents certain difficulties. In particular, the majority of the plant bioactive ingredients are prone to degradation. Factors such as light, temperature, oxygen, pH as well as the presence of metal ions during processing, storage or upon gastrointestinal digestion can influence their stability. Moreover, the poor aqueous solubility of hydrophobic compounds prevents their dissolution and absorption. In the same frame, the limited diffusion and permeability of such compounds across intestinal epithelium cells affect their bioavailability (i.e., the amount of an ingested bioactive compound that is absorbed in the gut after digestion) [2]. In the case of essential oils, their high volatility further limits their applications.

**Figure 2:**
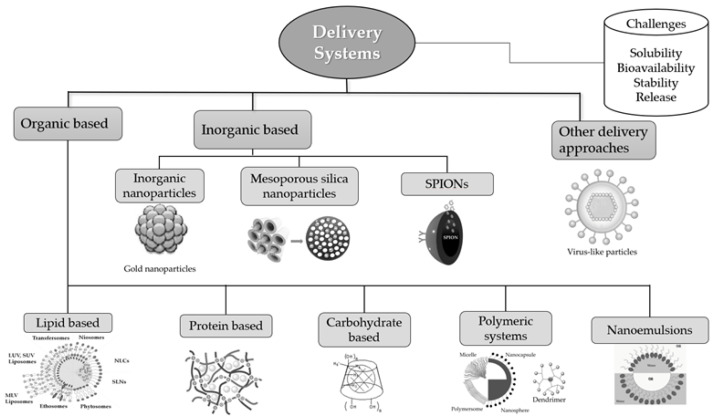
Types of carriers for the delivery of phytochemicals (abbreviations: SPIONs, superparamagnetic iron oxide nanoparticles; MLV, multilamellar vesicles; LUV, large unilamellar vesicles; SUV, small unilamellar vesicles; NLC, nanostructured lipid carriers; SLN, solid lipid nanoparticles).

The above-mentioned drawbacks can often be overcome by encapsulating the bioactive ingredients in appropriate delivery systems. Such systems offer various advantages including increased processing and storage stability, enhanced bioavailability, controlled release, effective delivery of the bioactive ingredients to specific sites-of-action where they can exert their activity promoting human health and well-being, masking of undesired flavors, incorporation of the bioactive ingredients into matrices without affecting quality characteristics (e.g., color), etc. [3]. Different types of carriers, i.e., organic (such as lipids (e.g., fatty acids, phospholipids), proteins (e.g., caseins, whey proteins, gelatin), carbohydrates (e.g., starch, cellulose, chitosan, pectin)), inorganic (e.g., silver, gold, mesoporous silica) and others (e.g., inactive viruses), have been used during the last decades in order to develop a wide range of delivery systems (e.g., nano- and microparticles, liposomes, hydrogels, nanoemulsions, nanofibers, etc.) employing a variety of encapsulation techniques (e.g., coacervation, electrospinning, emulsification, etc.) (Figure 2) [4,5,6,7].

The current review provides an overview of the latest advances regarding the design of innovative delivery systems for plant-derived bioactive compounds, extracts and essential oils in order to overcome the above-mentioned challenges. Moreover, a broad spectrum of food, cosmetic and medicinal applications, along with future trends, are critically discussed.

## 2. Challenges in the Use of Plant Bioactive Ingredients

Some of the most common challenges related to the exploitation of plant bioactive ingredients in food, cosmetic and pharmaceutical applications that are usually encountered are summarized below.

### 2.1. Solubility

One of the major challenges that limit the direct incorporation of plant bioactive ingredients into foodstuffs, beverages and cosmetic and pharmaceutical products is their low water or oil solubility. Compounds with poor water solubility (e.g., carotenoids) cannot readily be incorporated into aqueous-based products whereas ingredients with poor oil solubility cannot easily be incorporated into oil-based products [1].

### 2.2. Bioavailability

A critical aspect regarding the fabrication of novel systems for the delivery of plant bioactive ingredients is the increase of their bioavailability. The latter one depends on several factors such as the molecular and physicochemical characteristics of the bioactive compound, the interactions with other ingredients, its solubility, its stability upon gastrointestinal digestion conditions, etc. These are the reasons why many plant ingredients exhibit low bioavailability along with poor pharmacokinetics and low accumulation in the target tissues [8,9,10,11,12]. In this regard, the targeted design of carriers provides the possibility to overcome the above-mentioned limitations and enhance the bioavailability of plant ingredients, leading to more pharmacologically active formulations with increased stability, solubility and pharmacological activity as well as lower toxic effects [12]. This can be achieved through the control of the particle size of the fabricated delivery system, their surface properties and the release of the encapsulated ingredient(s) toward site-specific activity at an appropriate rate and dose [13]. Depending on the physicochemical nature and the chemical profile of the plant molecules to be encapsulated, several kinds of formulations have been used to increase their bioavailability, with lipid-based systems and especially liposomes being the most common ones. On the other hand, non-liposomal approaches include polymeric nanoparticles [14], nanoemulsions [15,16,17], quantum dots [18], micelles [19] and solid lipid nanoparticles.

### 2.3. Stability

Stability constitutes another important factor that may limit the food, medicinal and cosmetics applications of plant-derived ingredients, in the form of either isolated compounds, extracts or essential oils. Such compounds are prone to degradation (e.g., oxidation, hydrolysis, crystallization, enzymatic deterioration) during processing or storage or under gastrointestinal digestion conditions. Factors such as oxygen, temperature, pH, the presence of additives, metal ions, etc., influence their stability [20]. Encapsulation offers protection and increases the stability of various bioactive compounds. In this regard, nanoemulsions, liposomes, phytosomes, ethosomes and nanoparticles have been used for the increase instability of plant ingredients such as zedoary turmeric oil [21], verbascoside [22] and tea polyphenols [10].

### 2.4. Release

The delivery system has to be fabricated in such a way that it allows the release of the active ingredients at a specific site of action, at a controlled rate or as a response to a particular environmental trigger (e.g., pH, temperature). This trigger could take place either during food storage (e.g., release of an antimicrobial) or in the human body (e.g., release in the mouth, stomach, small intestine or colon) [1]. Release followed by carrier biodegradation are often important aspects for the design of an effective delivery system. The release mechanism of plant metabolites from a carrier involves (a) the desorption of metabolite(s); (b) the diffusion through the matrix (followed by diffusion through the carrier wall in some cases, e.g., nanocapsules); (c) the matrix decomposition; and (d) the combined decomposition/diffusion [23]. In sustained-release formulations, the metabolite(s) is released from the carrier at a continuous rate. This not only simplifies the application but also offers predictable and reproducible pharmacokinetics [24]. Furthermore, when referring to complex systems such as plant extracts or essential oils, rather than isolated metabolites, the term synchronized release may apply. It refers to the controlled release of multiple metabolites in a specific time frame while maintaining the inter-component ratio. This approach is of importance in such systems as the different physicochemical characteristics of the plant metabolites lead to asynchronous and non-controllable release, which usually causes decreased bioactivity [25]. Systems that have been used so far for the controlled release of various bioactive compounds include liposomes [26,27], nanoemulsions [28,29], polymers, etc. [30,31,32].

## 3. Organic-Based Delivery Systems

### 3.1. Lipid-Based Delivery Systems

**Figure 3:**
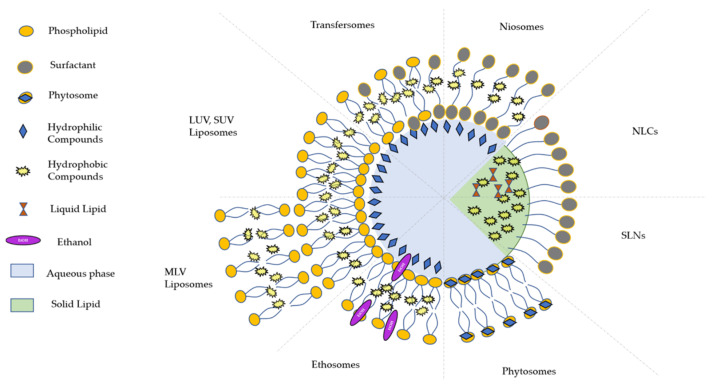
Schematic overview of the different lipid-based delivery systems (abbreviations: MLV, multilamellar vesicles; LUV, large unilamellar vesicles; SUV, small unilamellar vesicles; NLC, nanostructured lipid carriers; SLN, solid lipid nanoparticles).

Lipid-based nanosystems represent the largest and most investigated category of nanocarriers. Many formulations based on lipidic structures have been prepared, i.e., liposomes and similar carriers such as ethosomes, transfersomes, solid lipid nanoparticles, nanostructured lipid carriers, lipid drug conjugates, etc. [33]. Usually, these carriers demonstrate lower toxicity profiles and more reasonable cost compared to polymeric carriers [34]. They also exhibit specific desirable characteristics, such as the possibility to encapsulate both lipophilic and hydrophilic molecules, significant encapsulation efficiency, controlled release, biodegradability, ease of production, high bioavailability, suitability for administration via various administration routes (oral, intravenous (i.v.), topical, pulmonary, etc.) and targeted delivery through peripheral group modification. Moreover, they may be prepared by sustainable processes [35]. A schematic overview of the different lipid-based delivery systems is given in Figure 3.

Several lipids can be used for the preparation of lipidic nanosystems. Their desired characteristics include biodegradability, biocompatibility, stability, capability to produce nanosize particles with a low polydispersity index, high loading capacity and lack of toxicity [36]. The lipids that are commonly used for such purposes belong mostly to the triglyceride, partial glyceride, fatty acid and sterol categories. The major criteria for lipid choice deal with the physicochemical nature of the ingredient(s) to encapsulate and the desired characteristics of the formulation, such as ideal particle size, release profile, targeted delivery, route of administration and production cost.

The production methods vary. Some examples are electrospinning, gelation, layer-by-layer deposition, extrusion and emulsification.

#### 3.1.1. Vesicular Systems

Vesicular carriers are highly ordered systems that consist of concentric bilayers formed as a result of the self-assembly of amphiphilic building blocks [37]. Such systems can play a major role in the transport and targeting of encapsulated materials. The first developed and most investigated category is liposomes, while there is constant research on newer systems that are able to carry and provide desired characteristics to plant ingredients. Encapsulation of such ingredients in vesicular structures may stabilize, protect and prolong their presence in the systemic circulation, while possible toxicity may be reduced [38].

##### Liposomes

The oldest vesicular system, first developed in the 1960s, is liposomes (from the Greek words “lipos” (fat) and “soma” (body)) [33]. Liposomes are highly efficient and relatively easy to produce, have a size that ranges from a few nanometers to several micrometers and demonstrate specific advantages for the encapsulation and targeted delivery of both hydrophilic and lipophilic molecules. The main components of a liposomal formulation are phospholipids or sphingolipids. In many cases, sterols, such as cholesterol, and polymers are also used.

Several methods have been developed for liposome preparation [39]. In all of them, temperature must be maintained above the lipid phase transition temperature. Conventional preparation techniques include hydration, sonication and microemulsification, while newer, more efficient techniques have been also developed (e.g., the heating method, the osmotic shock method, spray drying, freeze drying, membrane-conductor method) [39].

Liposomal vesicles are usually classified based on the diameter and number of layers as multilamellar vesicles (MLVs), i.e., those that have multiple bilayers, and unilamellar vesicles, i.e., those that have a single bilayer. The latter ones can be further classified into large unilamellar vesicles (LUVs) and small unilamellar vesicles (SUVs) [40]. In both categories, the bilayer(s) enclose an aqueous core.

The main disadvantage of liposomal carriers is the production cost because usually lipidic raw materials are relatively expensive. Another main issue is the thermodynamic stability of such systems. Liposomes are prone to fusion, aggregation and unintended/premature release of the encapsulated ingredient(s). Moreover, the lipids are susceptible to oxidation phenomena. Finally, in the case of multiple molecule encapsulation, as in the case of plant extracts or essential oils encapsulation, interaction between incorporated ingredients at the liposomal interior is possible [41]. On the other hand, liposomal formulations demonstrate specific advantages, including lack of toxicity, flexibility, biocompatibility, biodegradability and non-immunogenicity [42]. 

The constant scientific research and the continuous demand for more evolved encapsulation nanosystems have led to advances in liposomal technology that can be categorized into four generations, according to their function [41]:

1. First generation of liposomes. These are the oldest developed, conventional liposomes that consist mainly of natural phospholipids and, in some cases, cholesterol. Despite the fact that they demonstrate a series of issues, such as increased uptake by the reticuloendothelial system (RES) and physicochemical and chemical degradation [43], they are very common delivery systems, also for plant ingredients [44,45].
2. Second generation of liposomes. The second generation includes more recent developments such as stealth and stimuli-responsive liposomes. Stealth liposomes are coated by polymers for the modification of size and charge. Polyethylene glycol-covered (PEGylated) liposomes improve the stability and reduce the probability of RES uptake, increasing the blood half-life of the system. Stealth liposomes with interesting properties have been developed in order to encapsulate resveratrol [46] and curcumin [47]. Stimuli-responsive liposomes are able to release their content depending on external triggering mechanisms, such as pH or temperature change, thus being more targeted than conventional ones. Resveratrol and curcumin have also been incorporated into pH-sensitive systems [48,49].
3. Third generation of liposomes. These systems bear a ligand (enzyme, antibody, vitamin, etc.) that leads to targeted transportation of the incorporated molecule(s) due to affinity mechanisms. Upon careful design, this can lead to accumulation of liposomes and targeted release at the desired site [50]. Galangin-loaded liposomes have been designed to target liver tissue [51], while a curcumin liposomal system has been developed to target cancer cells [52].
4. Fourth generation of liposomes (or theranostic liposomes) combine several strategies to achieve site-specific delivery and, at the same time, imaging [53]. Their main advantage is the multifunctionality—being diagnostic and therapeutic agents at the same time. For the moment in what concerns plant extracts, the literature is very limited. A good case study is the one by Wang et al. [54], who developed a magnetic targeting liposomal nanocarrier, loaded with resveratrol, that with the aid of an external magnetic field can cross the blood–brain barrier and could prove helpful for the treatment of cerebral disease.

Since their first appearance in the literature, liposomal systems have been widely investigated for the incorporation of plant ingredients. Liposomal incorporation has been proved to tackle several issues associated with natural products, such as low bioavailability, solubility and instability, as well as to provide desired characteristics including targeted delivery and controlled release rate. Plant ingredients for liposomal incorporation may be crude or fractionated extracts, essential oils or isolated compounds.

One of the most studied categories of metabolites to be encapsulated in liposomal formulations is polyphenols—plant secondary metabolites with many applications in human health. Polyphenols demonstrate several challenges, mainly instability and low bioavailability, which are the reasons for liposomal encapsulation [55]. In particular, quercetin, a flavonol found mostly in onions, grapes, berries, cherries, broccoli and citrus fruits, is one of the most investigated polyphenols for liposomal incorporation because of its diverse bioactivity that ranges from anti-inflammatory to anticancer [56]. Cellular protective effects of liposomes against oxidative stress were reported for quercetin-loaded liposomes [57]. The study also found enhanced internalization by cells for the liposomal system. In another study [55], liposomal encapsulation of quercetin led to very high 2,2-diphenyl-1-picrylhydrazyl (DPPH) scavenging and lipid peroxidation inhibition capacity, along with desired stability characteristics. High antioxidant capacity, through various techniques including DPPH, was proved by Hao et al. [58] for chitosan-coated quercetin-loaded liposomes. The system also presented high stability, solubility and biocompatibility. Quercetin has also been co-encapsulated with temozolomide in a liposomal system that proved effective in the treatment of glioma [59]. In another study utilizing tumor-bearing mice, quercetin-loaded liposomes treatment reduced the tumor growth compared to its free form [60]. The system did not cause any adverse effects in the liver and kidney of the mice. Curcumin is a polyphenolic compound isolated from *Curcuma longa* (turmeric) rhizomes. It exhibits significant bioactive properties such as chemopreventive, anti-inflammatory and anticancer, while its use is limited because of stability, low solubility and bioavailability issues. Thus, liposomal systems have been developed by several groups in order to increase the molecule’s health benefit potential. Compared to free curcumin, liposomal systems have been proved to provide enhanced anti-inflammatory activity, sustained-release properties and increased antioxidant activity [61,62]. Kianvash et al. [63] studied curcumin-propylene glycol-loaded liposomes for burn healing applications. The formulation caused the significant recovery of burned rat skin in 8 days—a result very similar to a potent silver sulfadiazine cream. Liposomal curcumin demonstrated better hepatoprotective effects against dimethylhydrazine-induced hepatic dysfunction in mice compared to free form and cyclodextrin complexed curcumin [64]. Liposomal curcumin has also provided some promising results against many types of cancer such as skin cancer [65,66], liver cancer [67], lung cancer [68,69] and brain cancer [70,71]. Resveratrol is one of the most studied stilbenes due to its pharmacological activities, which include antioxidant, anti-aging, anti-inflammatory, antidiabetic, cardioprotective, anticancer and neuroprotective properties [72]. The common polyphenol problems of instability, bioavailability and solubility limit its health applications. Resveratrol-loaded PEGylated liposomes were prepared by Caddeo et al. [46]. The study revealed long-term stability and biocompatibility as well as enhanced protection against oxidative stress in ex vivo human erythrocytes. In another study, transferrin-targeted resveratrol liposomes were prepared for the treatment of glioblastoma [73]. The results demonstrated a significant therapeutic effect, which was also increased compared to non-targeted resveratrol liposomes. Chitosan-coated liposomes were prepared to improve resveratrol’s topical delivery [74]. The in vitro study proved that resveratrol-loaded liposomal formulations can significantly improve the antioxidant and anti-inflammatory activity in comparison with free resveratrol. *Orthosiphon stamineus* exhibits significant bioactive properties such as diuretic, hepatoprotective, anti-angiogenic and anticancer [75,76,77], which have limited applications because the plant’s constituents have low water solubility. Aisha et al. [78] prepared nanoliposomes incorporating the plant’s ethanolic extract, resulting in enhanced solubility, absorption and finally antioxidant activity compared to the non-encapsulated plant. Similarly, tea polyphenols have been proved to benefit from liposomal encapsulation, by gaining enhanced bioavailability, stability and controlled release properties [9,79,80,81]. Liposomes have been used, among other targets, for the increase of bioavailability of *Panax notoginseng* saponins [82,83]. The saponins are poorly absorbed from the digestive tract when administered orally, probably because of decomposition in the stomach and low membrane permeability (attributed to high hydrophilicity and high molecular weight). Zhang et al. [82] developed a core-shell hybrid liposomal system that incorporated *P. notoginseng* saponins, increasing their bioactivity after oral administration in rats.

Besides isolated metabolites, liposomes loaded with various extracts have been also prepared. In particular, liposomes loaded with anthocyanins from a hibiscus extract (*Hibiscus sabdariffa*) have been fabricated [84]. The encapsulation efficiency was found to be high, and in parallel, the extract enhanced the stability of the lipids against oxidation. *Asparagus racemosus* root extract has also been incorporated into liposomes to assess the anti-inflammatory activity in the monocytic leukemia cell line THP 1 [85]. The system was found to be effective for topical and/or transdermal anti-inflammatory applications. Manconi et al. [86] encapsulated a polyphenol-rich grape pomace extract in liposomes associated with polymers for oral delivery. The system exerted antioxidant properties in Caco-2 cells while it resisted the low gastric pH. Pomegranate peel extract along with collagen hydrolysate, and shrimp lipid extract were encapsulated in liposomes that were subjected to freeze drying and subsequent incorporation in squid surimi gels. The gel proved to be stable—a fact attributed to the liposomes—while the antioxidant activity was maintained after in vitro gastrointestinal digestion [87]. Liposomes containing *Psidium* extracts have also been examined for their hepato-protective antioxidant activity in rats. Liposomal *Psidium guajava* leaves exhibited significant restorative properties in the liver tissue compared to silymarin [88]. In a different application, Pinila and Brandelli [89] incorporated nisin and garlic extract into a liposomal formulation that was proved to inhibit the growth of *Listeria monocytogenes*, *Staphylococcus aureus*, *Escherichia coli* and *Salmonella enteritidis* in milk, exhibiting potential as a natural antimicrobial agent and preservative.

Several studies have demonstrated the increased antimicrobial potential of liposome encapsulated essential oils. Liposomal *Zataria multiflora* essential oil exhibited lower minimum inhibitory concentration than free essential oil against *E. coli* [90]. In a study by Gortzi et al. [91], the antimicrobial activity of liposomal *Origanum dictamnus* extracts was found to be higher compared to the free form. Thymus essential oil has also been incorporated into liposomes to study the antimicrobial activity against *Streptococcus mutans* and *Candida albicans*. The results were promising for the treatment of caries [92]. 

##### Transfersomes, Ethosomes, Phytosomes and Niosomes

Since the 1990s, newer vesicular lipid vesicles with interesting properties have been developed. By chronological order, the main categories are transfersomes, ethosomes, phytosome and niosomes [93].

In particular, *transfersomes* are deformable vesicles that find mainly skin applications as their elastic properties favor transdermal penetration by either intracellular or transcellular route [94]. They have a liposomal structure, with the use of surfactants being the major difference. Surfactants provide flexibility and act as membrane-softening and destabilizing agents [95]. A transfersome carrier loaded with caffeine and minoxidil has been developed for the treatment of alopecia by Ramezani et al. [96]. The results showed an increase in hair length and weight in vivo that was attributed to improved encapsulation efficiency, release rate and stability. Transfersomes, loaded with apigenin, have demonstrated enhanced stability, permeability and prolonged release characteristics [97]. Moreover, a system consisting of epigallocatechin-3-gallate (from *Camellia sinensis*) and hyaluronic encapsulated in transfersomes exhibited improved in vitro solubility and stability as well as ex vivo skin permeability along with antioxidant and anti-aging properties [97].

*Ethosomes* have been developed as effective vesicles for topical, transdermal and systemic applications. They are formed from phospholipids, water and high concentrations of alcohols (ethyl alcohol or isopropanol) [98] that provide elastic properties to the vesicles as well as increase the encapsulation efficiency of lipophilic molecules, especially compared to conventional liposomes. Halan et al. [99] prepared ethosomes incorporating caffeic acid for transdermal delivery. The study revealed that the encapsulation efficiency of caffeic acid was greater compared to other systems described in the literature and that caffeic acid was stabilized, maintaining its antioxidant potential for prolonged time. Ginsenoside Rhl derived from *Panax ginseng* has been also incorporated into ethosomes [100]. Enhanced skin permeation, retention and deposition in vitro using human cadaver skin was observed, even though transfersomes were found to be superior for this application.

*Phytosomes* are produced by the interaction of metabolites contained in plant extracts with phosphatidylcholine. The formulations exhibit good solubility and bioavailability properties [101]. Isolated compounds (e.g., silymarin, curcumin) as well as extracts (e.g., milk thistle, green tea, grape seed, *Ginkgo biloba*) have been complexed in phytosomes exhibiting enhanced properties compared to free forms [102].

*Niosomes* are surfactant vesicles which are made of synthetic non-ionic surfactants and lipids, mainly cholesterol [103]. Niosome structure, which is essential for its pharmacokinetic properties, depends on several parameters, such as the temperature of lipids hydrations, type and concentration of surfactant and method of preparation [104]. Plant ingredients may benefit from niosome formulations as the latter may provide increased solubility, resulting in higher bioavailability, controlled release and stability. Innovative herbal niosome formulations appear to have beneficial properties in crossing the blood–brain barrier offering targeted delivery [104]. In particular, *Myrtus communis* extract has generally limited applicability due to its low solubility and permeability. However, upon its incorporation into stable multilamellar niosomes, it was found to exhibit higher antimicrobial activity than the free extract. The system also demonstrated in vitro release loading efficiency characteristics [105]. The lipophilic flavonoid morusin has been incorporated into niosomes, resulting in increased solubility and encapsulation efficiency, controlled release and anticancer activity against various types of cancer [106]. Similar results were described in a study regarding the encapsulation of the naphthoquinone lawsone, derived from henna [107]. Marigold extract [108], curcumin [109,110,111] and essential oils [112,113,114] are some of the plant-derived materials that have successfully been incorporated into niosomes.

Extracellular vesicles are a novel diverse category of delivery systems that are able to efficiently encapsulate natural products [115]. Microparticles, exosomes and apoptotic bodies isolated from different cell types have been investigated as their engineered versions seem to enhance the bioavailability and stability of plant ingredients. In particular, microparticles range between 100 and 1000 nm and are generated by plasma membrane blebbing. Exosomes, which are further categorized as small exosomes, large exosomes and exomeres, originate from the endosomal system and range from 30 to 150 nm [116]. Apoptotic bodies have a size range of 50 to 5000 nm and are generated by plasma membrane blebbing during apoptosis [115,117].

Exosomes derived from cells treated with curcumin and epigallocatechin gallate have been shown to increase polyphenol bioactivity against cellular models of disease such as reverses LPS-induced pro-inflammatory gene expression in buffalo granulosa cells [118], increases exosomal TCF21, thus suppressing exosome-induced lung cancer [119], etc. [118,119,120,121,122,123]. Exosomes have also been investigated for their ability to incorporate curcumin, acting as delivery systems. The results seem very promising in terms of efficacy and bioavailability enhancement [124,125,126,127]. Anthocyanins, cyanidin, delphinidin, petunidin, peonidin and malvidin have also been shown to benefit from exosome encapsulation [128,129] in terms of efficacy.

#### 3.1.2. Νon-Vesicular Systems

Non-vesicular delivery systems include solid lipid-based nanocarriers. They are colloidal particles with size that ranges from 50 to 1000 nm [130] and are produced using various methods either of high energy, such as high-pressure homogenization, high-speed homogenization, emulsification–evaporation and ultrasounds, or of low energy, e.g., utilizing solvents and microemulsion/double emulsion techniques [131]. Due to their versatility, non-vesicular lipid-based nanocarriers have been studied for a variety of medicinal applications such as gene transfer, bioimaging, antimicrobial activity, etc. Being highly biocompatible, they can be administered via various routes, e.g., orally, intravenously, topically [130]. Various plant isolated compounds as well as extracts have been encapsulated in such delivery systems.

##### Solid Lipid Nanoparticles

*Solid Lipid Nanoparticles* (SLNs) are colloidal carriers produced by adding non-ionic emulsifiers to stabilize the dispersion of melted (at room and human body temperatures) solid lipids in water. Large surface area, high encapsulation efficiency, controlled release and targeted delivery are some of the characteristics that have made SLNs one of the most investigated nanocarriers during the last years [132]. SLNs demonstrate several advantages: in particular, the lipophilic matrix they provide allows the encapsulation of a wide range of compounds of different lipophilicity. Moreover, SLN formulations may improve plant ingredient stability and reduce possible adverse effects [3], they do not demonstrate toxicity, they are highly biodegradable, and they have a wide flexibility in terms of size, surface functionalization as well as increased cellular uptake. They can be also easily produced on a large scale and yield solid final formulations that enhance stability and facilitate industrial logistics issues [133].

SLNs are categorized into three types: Type I is the homogeneous matrix model, where the bioactive compound(s) is dispersed in the lipid core, Type II is called the drug-enriched model, where a drug-free lipid core is formed and an exterior solid shell contains both lipid and the bioactive compound, and Type III is the drug-enriched model, where the bioactive compound(s) concentration is close to its saturation solubility in the lipid. This causes its precipitation in the core and a lipid cover is formed. Slight changes in the manufacturing process may alter drastically the functionality of SLNs of all types, making them very versatile [134].

SLNs have been widely investigated for the encapsulation of plant bioactive compounds. In particular, cationic SLNs loaded with epigallocatechin-3-gallate (EGCG) have been studied for the activity against different cell lines (i.e., Caco-2, HepG2, MCF-7, SV-80 and Y-79). EGCG produced concentration- and time-dependent antiproliferative effects, depending on the cell line, while toxicity/biocompatibility issues were raised [135]. SLNs from cocoa butter and surfactants have also been loaded with EGCG for food applications. The stability of EGCG was found to be enhanced while controlled release was achieved [136]. In a similar system, the pharmacokinetic parameters of EGCG were significantly improved, based on bioavailability, stability and controlled release studies [137]. SLNs have also been fabricated by the use of tristearin and polyethylene glycol (PEG)ylated emulsifiers for the encapsulation of curcumin aiming at increasing its oral bioavailability. Indeed, the bioaccessibility of curcumin was found to be increased under gastrointestinal digestion conditions [138]. Moreover, the oral bioavailability of curcumin loaded in long-PEGylated SLNs was found to be increased [138] and depended on the type and concentration of the emulsifier. Enhanced solubility, stability, permeability and bioavailability have also been reported for curcumin-loaded SLNs [139]. The authors suggested that these properties could be exploited for anti-inflammatory and anticancer applications. In the same frame, the oral absorption of quercetin-loaded SLNs has also been investigated employing in situ perfusion in rats [140]. Its bioavailability was found to be 5-fold higher compared to that of the free molecule. SLNs loaded with resveratrol have also been reported in literature. In particular, resveratrol-loaded stearic acid-based SLNs have also been orally administered in Wistar male rats. This lipid formulation was found to improve the oral bioavailability of resveratrol compared to that of a resveratrol suspension [141]. Moreover, resveratrol-loaded SLNs have been found to inhibit cardiotoxicity associated with the administration of the anticancer agent doxorubicin in mice [142]. SLNs using *Theobroma grandiflorum* seed butter have also been prepared for topical applications of resveratrol. The results revealed increased antioxidant activity, permeation and retention of resveratrol in the human skin, as well as controlled release [143]. Resveratrol has been also loaded in SLNs in a study against insulin resistance through improving the hypoglycemic effect and up-regulating the expression of diabetes-related proteins. The formulation was administered to rats and the results revealed promising hypoglycemic properties [144]. SLNs loaded with (+)-limonene 1,2-epoxide have also been produced using glycerol monostearate by means of hot high-pressure homogenization [145]. The prepared SLNs were found to ameliorate lipid peroxidation and cytotoxicity in the spontaneously transformed aneuploid HaCaT keratinocyte cell line from adult human skin.

Apart from isolated compounds, plant extracts have been encapsulated in SLNs as well. In particular, a pomegranate extract, containing ≥30% punicalagin, was encapsulated in SLNs. The formulation procedure was optimized in terms of various parameters including lipid and surfactant type and concentration, co-surfactant concentration, sonication time, particle size, polydispersity index, zeta potential, entrapment efficiency and cumulative drug release [146]. Additionally, SLN functionalized anti-transferrin receptor monoclonal antibodies were loaded with grape seed and skin extracts. Extracts were found to be more effective on the inhibition of Aβ(1–42) fibril formation compared to isolated resveratrol. Experiments on human brain-like endothelial cells demonstrated that the cellular uptake of functionalized SLNs was more efficient than that of non-functionalized ones and that SLNs that were functionalized with an unspecific antibody could potentially find application in Alzheimer’s disease treatment.

A variety of essential oils has also been encapsulated in SLNs [147]. In particular, SLNs loaded with *Z. multiflora* essential oil have been prepared [148]. The obtained delivery system demonstrated higher in vitro antifungal activity than the free essential oil. SLNs have also been loaded with Yuxingcao essential oil aiming at pulmonary sustained delivery. Upon nebulization, the obtained SLN systems exhibited in vitro reparability and appeared to extend the essential oil retention as well as to improve pulmonary availability [149]. In another study, SLNs were used for the encapsulation of Peppermint essential oil. Even though this system showed promising results related to gastrointestinal health and antimicrobial capacity, the authors suggested that its application is limited due to its strong odor [150].

##### Nanostructured Lipid Carriers

*Nanostructured lipid carriers* (NLCs) constitute colloidal delivery systems, similar to SLNs with the difference that they are composed of a mixture of solid and liquid lipids. This leads to the formation of an unorganized core matrix that is covered by one or more surfactants [151]. The low ordered matrix prevents early compound release and achieves high encapsulation efficiency. This fact, in combination with the good biodegradability and biocompatibility of the lipids used, constitute NLCs a highly advantageous system for the enhancement of stability, loading and controlled release of plant ingredients [152]. Three morphological types of NLCs have been described in literature: NLC type I (imperfect crystal model), which is described by high loading efficiency but no sustained release due to highly disordered lipid matrix; NLC type II (multiple type) or oil/lipid/water type, which offer both high loading capacity and controlled release; and NLC type III (amorphous model), which is described by the creation of an amorphous lipid matrix of high homogeneity based on the choice of lipids [151,153].

NLCs have been prepared for the encapsulation of silymarin, which exerts low bioavailability due to its lipophilicity. After its loading in the NLCs, the absorption of silymarin was increased and it demonstrated physicochemical stability [152]. Moreover, quercetin has been loaded in NLCs and SLNs for brain delivery and the results demonstrated higher loading efficiency and bioavailability for the NLC formulations [154]. For the enhancement of cell penetration of curcumin in photodynamic therapy of cancer, NLCs have been prepared. Increased anticancer activity was noticed under both dark and light conditions [155]. In vitro digestion and release studies were performed for curcumin-loaded NLCs that were found to exhibit controlled release while the system was found to be stable under the simulated digestion conditions for 2 h [156]. For the increase in the (low) antiplasmodial activity of free curcumin due to its low bioavailability, NLCs were developed and the results were found to be promising for the treatment of malaria [157]. Partially hydrolyzed ginsenoside was used for the modulation of the in vitro release and bioavailability of curcumin-loaded NLCs [158]. *H. sabdariffa* extract was incorporated into NLCs and quercetin and anthocyanins were entrapped in high concentration while the systems developed were physicochemically stable. Furthermore, various essential oils have been encapsulated in NLCs toward their protection and increased bioavailability [159,160,161,162].

An overview of the different lipid-based delivery systems that have been employed for the encapsulation of either pure plant bioactive compounds, extracts or essential oils, is given in Table 1.

### 3.2. Protein-Based Delivery Systems

Apart from their nutritional value, proteins are macromolecules that, based on their biocompatibility, biodegradability, their ability to self-associate as well as their emulsifying, foaming and gelation properties, are appropriate candidates for the encapsulation of both hydrophilic and hydrophobic bioactive ingredients such as phenolic compounds, carotenoids, polyunsaturated fatty acids, vitamins, etc. A wide variety of protein-based delivery systems, including hydrogels, micro- and nanoparticles, films, etc., have been reported in literature. Various approaches have been used for their formulation such as coacervation, i.e., a process based on the electrostatic attraction between oppositely charged groups of different biopolymers (e.g., a protein and a carbohydrate), cold gelation, i.e., a process used to form protein-based hydrogels at ambient temperature involving various steps, spray drying, electro-hydrodynamic processes (e.g., electrospinning and electrospraying) that are based on the use of electrical charges to produce fibers and particles, as well as the antisolvent precipitation method that is used to produce protein nanoparticles [166]. The proteins that are most commonly used as wall materials for the design of food-grade delivery systems can be either of animal origin, e.g., casein, whey proteins, gelatin or of plant origin such as those obtained from soy, cereals (e.g., zein) and legumes (e.g., pea) [167].

Regarding proteins of animal origin, caseins (αs1, αs2, β and κ types), the major milk proteins (~80% of total protein content), have gained attention as carriers for various bioactive compounds based on their natural tendency to self-assemble as spherical colloidal nanoforms, namely micelles, as well as on their emulsifying and stabilizing properties [167]. Indeed, casein-based nanoparticles and re-combined casein micelles have been prepared for the delivery of epigallocatechin gallate and folic acid [168]. The authors suggested that the encapsulation of these molecules resulted in their increased stability against heat-induced degradation at 74 °C for 20 s. The encapsulation of resveratrol in casein nanoparticles prepared by a coacervation process followed by spray drying has also been examined [169]. Upon oral administration of the resveratrol-loaded casein nanoparticles to rats, its bioavailability was found to be ten times higher compared to that after its administration as an oral solution. The encapsulation of β-carotene via hydrophobic interactions in aggregated casein and re-assembled casein micelles has been also reported [170]. The encapsulated β-carotene showed enhanced stability upon storage at 11% and 75% relative humidity for 21 days. The preparation of re-assembled casein particles loaded with vitamin D has also been investigated [171]. The authors concluded that encapsulated vitamin D was found to be more stable during storage for 42 days at ambient temperature compared to the control. Re-assembled casein micelles and casein nanoparticles have also been prepared for the encapsulation of the hydrophobic compounds quercetin and curcumin [172]. After encapsulation, their aqueous solubility was found to be higher than that of the respective free molecules, whereas they also exhibited cytotoxic effects against the MCF-7 breast cancer cell line. 

Regarding the other major milk protein representatives, i.e., whey proteins, which are derived from whey, a by-product of cheese production, they have also received growing attention for the preparation of delivery systems based on their safety, low cost as well as gel-forming and emulsifying properties. They are composed of various globular proteins such as α-lactalbumin, β-lactoglobulin, bovine serum albumin, etc. The most widely known whey protein products are the whey protein concentrates (WPCs) that are obtained via the ultrafiltration of whey and the whey protein isolates (WPIs) that are obtained after further processing including diafiltration of ion exchange [173]. Mixed hydrogels composed of whey protein aggregates prepared by cold gelation in the presence of k-carrageenan have been designed to protect curcumin under gastrointestinal digestion conditions [174]. The authors concluded that these gels prevented the degradation of curcumin in the upper gastrointestinal tract and may be suitable for its colon-specific delivery. Whey protein nanofibrils have also been used as carriers for curcumin by the same research group [175]. The nanofibrils were produced by heating (85 °C) whey protein isolate solution at pH 2.0 for 5 h. Loading of the formulated whey protein nanofibrils with curcumin was found to improve its aqueous solubility at acidic conditions (pH = 3.2), to decrease its sedimentation during storage for one month as well as to increase its in vitro antioxidant activity using the DPPH assay. Whey protein concentrates and whey protein isolates have also been employed as nanocarriers of curcumin [176]. The nanoparticles formulated using whey protein concentrate as wall material showed the highest antimicrobial activity against *Bacillus subtilis*, *S. aureus*, *Pseudomonas aeruginosa*, *E. coli* and *C. albicans* using the agar well diffusion method. On the other hand, the nanoparticles prepared using whey protein isolate exhibited the highest anticancer activity against the HepG2 cell line. In both cases the effects were found to be dose-dependent. Nanoemulsions using whey protein isolate or polymerized whey protein isolate loaded with lutein have also been prepared with the aid of high intensity ultrasounds [177]. The whey protein isolate-based nanoemulsion system was found to be stable during storage at 4 °C for four weeks with lutein being reduced by only 4%. Whey protein concentrate has also been used for the preparation of an oil-in-water emulsion containing flaxseed oil, a rich source of n-3 fatty acids, e.g., α-linolenic acid [178]. The obtained emulsion was found to be stable at 4–7 °C for 28 days with no indication of phase separation. Moreover, a lower increase in peroxide value (~21%), an indicator of oxidation, was observed for the flaxseed oil emulsions compared to that of the free oil (~44.3%). Apart from pure compounds, flavonoids from various citrus peel extracts have also been encapsulated in whey protein concentrate nanoparticles [179]. The authors suggested that the encapsulation delayed the release of flavonoids under in vitro gastrointestinal conditions, whereas their antioxidant activity was improved compared to the free citrus peel extracts. The encapsulation of spray-dried beetroot extract in whey proteins and certain oligosaccharides, i.e., maltodextrin and inulin, has also been reported [180]. It was shown that the simultaneous use of whey protein isolate and inulin resulted in enhanced stability and antioxidant activity of the beetroot extract powder upon storage at 60 °C. Whey proteins have also been employed for the encapsulation of various essential oils apart from pure compounds and extracts. In particular, whey protein isolate-based edible films loaded with thyme or clove essential oils have been formulated via homogenization [181]. The application of these films on Kashar cheese resulted in the reduction of *E. coli* O157:H7, *L. monocytogenes* and *S. aureus* counts after 60 days of storage at 4 °C. Moreover, whey protein isolate-based films loaded with oregano or garlic essential oils prepared employing homogenization were applied on sliced Kashar cheese [182]. The authors found that the examined cheese product exhibited microbial stability against *E. coli* O157:H7, *S. enteritidis*, *L. monocytogenes*, *S. aureus* and *Penicillium* spp. during storage. 

The acid or alkaline hydrolysis of the protein collagen, which is found in nature as the major constituent of skin, bones and connective tissue, results in another animal-based biopolymer, namely gelatin. The latter finds application as wall material for the development of delivery systems for pure bioactive compounds, plant extracts and essential oils [166]. Particularly, curcumin has been encapsulated in electrospun gelatin nanofibers [183]. The authors suggested that the use of cationic cetyltrimethyl ammonium bromide as a surfactant facilitated the release of curcumin, resulting in a higher in vitro radical scavenging activity (DPPH assay) and ferric reducing antioxidant power (FRAP assay), as well as a stronger antimicrobial activity against *S. aureus* compared to control nanofibers without curcumin. Cardamom extract-loaded gelatin nanoparticles have also been prepared by a two-step desolvation method [184]. The prepared nanoparticles were tested as a drug delivery system to treat glioblastoma and were found to effectively eradicate human U87MG glioblastoma cells. Regarding the encapsulation of essential oils, chitosan-gelatin coatings containing nano-encapsulated tarragon essential oil have been produced [185]. The obtained coatings were applied on pork slices during refrigerated storage for 16 days and were found to inhibit their quality deterioration, contribute to the sustained release of the tarragon essential oil and cause an improvement in antioxidant, antibacterial and sensory properties. Orange essential oil has also been loaded in electrospun gelatin and gelatin-cross-linked tannic acid nanofibers [186]. The results suggested that the prepared gelatin nanofibers provided controlled release of orange essential oil and improved its storage stability at 25 °C and 33% relative humidity.

Taking into account that the global population is expected to reach 9.5 billion by 2050, the animal protein demand is estimated to double during this period in order to cover the needs. Considering that the increasing production and consumption of animal proteins is linked with environmental concerns related to land and water requirements as well as greenhouse gas emissions, plant protein production that requires the consumption of less natural resources constitutes a sustainable alternative approach. In this view, the development of plant protein-based delivery systems could result in the production of novel functional foods, nutraceuticals, cosmetics, etc. of importance to certain consumer groups, e.g., vegans [187]. Regarding plant-based proteins, soy proteins have been employed for the fabrication of delivery systems for different bioactive compounds taking advantage of their gelation and emulsifying properties as well as their tendency to aggregate, etc. [188]. Apple and red grape pomace extracts rich in polyphenols have been encapsulated in soy protein nanocapsules [189] using a nanoemulsification process. Enhanced in vitro antioxidant activity was observed for the encapsulated extracts. Resveratrol has also been encapsulated in soy protein isolate nanoparticles using a rotary evaporation technique [190]. The obtained resveratrol-loaded nanoparticles exhibited more than two times higher solubility with significantly increased dissolution and stability compared to the unencapsulated resveratrol. Additionally, soy protein isolate nanoparticles loaded with curcumin have also been fabricated [191]. The formation of these nanoparticles enhanced the solubility of curcumin in water compared to the unencapsulated form and improved its storage stability and bioaccessibility as determined by in vitro simulated digestion experiments. Soybean protein isolate nanoparticles containing β-carotene have been prepared by the homogenization–evaporation method [192]. The cellular antioxidant activity of β-carotene in the obtained nanoparticles was found to be improved compared to the free molecule.

Besides soy proteins, zein is the major storage protein in corn, accounting for 35–60% of total proteins, and it is entirely found in endosperm. It has the ability to self-assemble in the presence of polar solvents, such as water, in order to form various structures as delivery vehicles for bioactive molecules [193]. For example, zein nanoparticles loaded with quercetin have been fabricated employing an antisolvent precipitation method [194]. In this way, the photochemical stability and the ABTS+ scavenging ability of quercetin were found to be enhanced. The same encapsulation method was also used for the preparation of curcumin-loaded zein nanoparticles [195] that were found to increase its bioaccessibility examined using an in vitro gastrointestinal digestion model. Zein nanoparticles loaded with curcumin and stabilized with dextran sulfate have also been prepared using an antisolvent precipitation method [196]. The authors concluded that curcumin loaded into the fabricated zein nanoparticles exhibited increased storage stability and bioaccessibility. Zein fibers loaded with curcumin have been prepared by the electrospinning technique as well [197]. The obtained curcumin-loaded fibers exhibited antibacterial activity against *S. aureus* and *E. coli* and the authors suggested that they could find use in active food packaging applications. Lutein has also been encapsulated in zein nanoparticles via solvent-induced nanoprecipitation [198]. The incorporation of lutein into zein nanoparticles resulted in its increased digestive stability, compared to aqueous lutein dispersions. The protection against chemical degradation as well as the controlled release of lutein after its encapsulation in zein nanoparticles stabilized with surfactants has also been reported [199]. Apart from zein nanoparticles, zein fibers have been prepared as well for the entrapment of various bioactive molecules. In particular, *Yerba mate* extract has been encapsulated in electrospun zein fibers [200]. Zein fibers loaded with 5% of this extract showed high antioxidant activity, greater thermal stability compared to the unencapsulated extract as well as the release of approximately 49% of extract within 50 h in a hydrophilic food simulant medium. Moreover, ribbon-like zein nanofibers containing Barije essential oil, known for its antidiabetic and antioxidant properties, have been prepared using the electrospinning technique [201]. The manufactured zein nanofibers showed α-glucosidase and α-amylase inhibition activity as well as release behavior into simulated stomach media described by a first-order model.

Pea proteins that are extracted from pea seeds consist of a mixture of different types of globular proteins e.g., globulins, albumins and glutelins. They possess gel-forming and emulsifying properties that make them suitable candidates for the fabrication of delivery systems either alone or in combination with various polysaccharides [166]. Pea protein–high-methoxyl pectin–rhamnolipid ternary complexes have been fabricated for the co-encapsulation of curcumin and resveratrol [202]. These complexes were found to retard light and thermal degradation of both compounds, provide a protective effect under gastric conditions and control their release in the intestine phase. The same research group has also fabricated ternary complexes composed of pea protein isolate, high-methoxyl pectin and individual surfactants such as rhamnolipid, tea saponin and ethyl lauroyl arginate for the delivery of resveratrol [203]. Pea protein isolate nanoparticles, fabricated with calcium-induced cross-linking, have been used as potential nanocarriers for protecting resveratrol from degradation, as well as improving its in vitro antioxidant activity [204]. The obtained complexes were found to retard photo- and thermal degradation of resveratrol as well as to delay its release during in vitro digestion. Encapsulation of quercetin in pea protein isolate and mesquite gum complexes has also been reported in the literature and resulted in its protection against UV degradation and its physical and chemical stability compared to free quercetin [205]. Pea protein nanoemulsions and nanocomplexes have been formed to protect cholecalciferol (vitamin D_3_) against UV radiation [206]. The authors suggested that the prepared nanostructures were found to increase the stability of cholecalciferol upon storage for 30 days as well as to enhance its recovery in micelles upon in vitro digestion. Moreover, a mixture of pea protein and maltodextrin as wall materials has been used for the encapsulation of rice bran oil [207] as well as black pepper seed oil [208]. Pea proteins have been used as wall materials also for the microencapsulation of propolis extract by spray drying [209]. The obtained microparticles exhibited improved thermal stability. A pea protein-modified starch complex has been used as wall material for the microencapsulation of canola oil containing docosahexaenoic acid (DHA) [210]. The utilization of this protein–polysaccharide complex resulted in the preservation and improvement of the oxidative stability of DHA during storage at room temperature for 30 days compared to the free oil. Moreover, conjugated linoleic acid (CLA) has been microencapsulated by spray drying in pea protein isolate, pea protein concentrate as well as their mixtures with maltodextrin and carboxymethylcellulose [211]. Encapsulated CLA was found to be stable at room temperature for 60 days, whereas the carbohydrate addition was not found to affect its stability. The design of lycopene-loaded oil-in-water emulsions stabilized by pea proteins has also been reported in literature [212]. The authors concluded that lycopene was found to be stable after 14 days of storage in a refrigerator.

In spite of the increasing number of publications related to protein-based delivery systems as well as their well-investigated role in enhancing the solubility, stability and bioavailability of a variety of plant-derived bioactive compounds, there are still challenges in this field. In particular, the majority of the relevant published studies have been carried out on a laboratory scale due to the lack of cost-effective methods to scale up production. In this regard, emphasis should be given to the development of large-scale production methods that, along with standardization, will assist in the commercialization of the formulated plant bioactive-loaded protein-based delivery systems [175]. 

### 3.3. Carbohydrate-Based Delivery Systems

Carbohydrates, along with lipids and proteins, are natural macromolecules that find applications as building blocks of delivery systems based on their unique characteristics, including water solubility, biocompatibility, biodegradability, binding ability via functional groups and molecular structure, which allow them to entrap a variety of hydrophilic and hydrophobic molecules. Moreover, carbohydrates are considered to be more thermally stable compared to lipid- and protein-based delivery systems, which can be melted or denaturated, respectively [213]. Taking into account all the above, carbohydrate-based delivery systems find numerous applications in food, pharmaceutical and cosmetic industries. Different carbohydrates have so far been exploited toward the preparation of delivery systems either in their natural or modified form after physical, chemical or enzymatic treatment [214].

In particular, starch, the most abundant storage carbohydrate in plants, is a biodegradable, biocompatible, low-cost biopolymer that consists of two macromolecules, namely amylose, which is linear, and amylopectin, which is branched. Starch finds numerous applications as an encapsulating material. Its hydrophilic nature, however, limits its use regarding the encapsulation of hydrophobic compounds, whereas another limitation in its use stems from its sensitivity to amylase activity that may begin to take place in the mouth. However, these drawbacks can be tackled through its modification using enzymatic (e.g., a-amylase), physical (e.g., extrusion,) or chemical (e.g., acid hydrolysis) methods [213] in order to extend its industrial applicability. Various starch-based delivery systems, including nanoparticles, nanocrystals and nanofibers, have been designed employing a variety of methods such as self-assembly, nanoprecipitation, ultrasonication, electrospinning, extrusion, microfluidization, etc. [215]. The partial hydrolysis of starch results in the production of other valuable polysaccharides, namely maltodextrins, which is more hydrophilic compared to starch. Maltodextrins are categorized by their dextrose equivalents (DE) that represent the amount of reducing sugars that is present in the molecule. Maltodextrins are biocompatible biopolymers that also find numerous applications as wall material in delivery systems of different bioactive compounds. Moreover, the enzymatic conversion of starch results in the production of cyclodextrins (CDs), which are cyclic oligosaccharides that typically contain six, seven or eight d-(+)-glucopyranose units (i.e., α-, β- and γ-CD, respectively) linked by α-1,4 glycosidic bonds. Cyclodextrins have a hydrophobic central cavity and a hydrophilic outer surface, a structure that allows them to form inclusion complexes with a variety of bioactive compounds via non-covalent forces (e.g., van der Waals forces, hydrogen bonds). The inclusion complexation is based on co-precipitation, which occurs after the addition of a guest molecule to a cyclodextrin aqueous solution upon stirring, sonication and/or heating. β-CD is the most commonly used among all cyclodextrins. It can be modified (e.g., hydroxypropyl-β-CD, hydroxyethyl-β-CD, methyl-β-CD) in order to tackle drawbacks related to low aqueous solubility, such as other polysaccharides [213,216]. It is worth mentioning that aqueous solutions of cyclodextrins have been used also as enhancers for the green extraction of polyphenols of a variety of plant materials, e.g., pomegranate fruit [217], *Sideritis scardica* [218], oak acorn husks [219], etc. Chitosan is a natural, non-toxic, biodegradable, biocompatible, cationic polysaccharide that derives from the alkaline deacetylation of chitin. The latter is the second-most abundant polymer in nature, after cellulose, and it is of low cost as it is obtained from marine waste. It possesses antimicrobial and antioxidant activities, whereas its mucoadhesive properties make it a good candidate as an absorption enhancer across intestinal epithelium for drugs, proteins, etc. Modified forms of chitosan can be prepared via three main reactions, namely depolymerization (e.g., acid hydrolysis, deamination), substitution (e.g., methylation, acylation) and chain elongation (e.g., cross-linking, graft copolymerization) toward improving its functional properties. The wide range of molecular weight and percentage of deacetylation of chitosan broaden its applications. A variety of carriers prepared with chitosan, such as nanoparticles, nanofibers and nanocomposites, have been reported in the literature employing different encapsulation approaches including nanoprecipitation, emulsion–ionic gelation, spray drying, etc. [220]. Another promising wall material is pectin, which is an anionic, water-soluble polysaccharide, naturally found in cell walls of plants. Its major sources are apple pomace and citrus peels as well as wastes derived from citrus processing. However, various plant materials have been used for pectin extraction, such as pomegranate peels, grapefruit peels, banana peels, mango peels, passion fruit peels, etc. [221]. Pectin consists of linear a-(1-4)-d-galacturonic acid units that are usually esterified. Based on the degree of esterification (DE), pectin can be divided into high-methoxyl pectin (HMP) (more than 50% DE) and low-methoxyl pectin (LMP) (less than 50% DE) [213]. It is worth mentioning that depending on the DE, pectin has a different hydrophobicity. In particular, high-methoxyl pectins are highly hydrophobic and can thus interact with hydrophobic molecules. Moreover, pectin is poorly absorbed in the upper gastrointestinal tract (i.e., mouth, stomach and small intestine), but it can be absorbed in the colon, after its digestion, by pectinolytic enzymes produced by colonic microflora. This makes it a suitable vehicle for colon-targeted bioactive compounds [222,223]. Various encapsulation techniques (e.g., nanocomplex formation, emulsification, spray drying) have been employed for the preparation of pectin-based vehicles such as nanohydrogels, nanoemulsions, nanoliposomes, etc. Gums constitute a class of hydrophilic polysaccharides that can interact with water to form viscous solutions, emulsions and gels. Considering their biodegradability, biocompatibility as well as the availability of reactive groups for molecular interactions, gums have been used as wall materials for the encapsulation of a variety of bioactive compounds. The most commonly used gums include gum Arabic, xanthan, carrageenan, etc., whereas gums from non-traditional sources, e.g., cress seed, basil seed, etc. (native gums) are also used considering their technological and functional properties (e.g., emulsifying, thickening) along with their low cost. Various gum-based structures, such as nanoparticles, nanofibers, nanocomplexes and nanoemulsions, have been fabricated using electrospinning, coacervation, antisolvent precipitation and emulsification techniques [224]. The most abundant polysaccharide on Earth that constitutes the major component of plant cell walls is cellulose, which also finds application as building block for delivery systems. Like other carbohydrates, cellulose can be physically, chemically or enzymatically modified to tackle some of its drawbacks including its low water solubility [213]. Cellulose nanocrystals as well as cellulose nanofibers have been fabricated as cellulose-based delivery systems. The preparation of the former ones involves several steps, including enzymatic or acid hydrolysis as well as mechanical treatment or oxidation, that aim at separating the amorphous domains of cellulose, which can derive from various sources (e.g., wood, cotton), and collecting the crystalline ones. The preparation of cellulose nanofibers requires the same steps as those for the preparation of nanocrystals, i.e., a mechanical treatment such as high-pressure homogenization, ultrafine friction grinding, cryocrushing, blending, etc., with or without a pretreatment step, e.g., acid hydrolysis, enzymatic fractionation, carboxymethylation, etc. The major difference between cellulose nanocrystals and cellulose nanofibers is that the former are exclusively of crystalline nature whereas nanofibers are composed of both amorphous and crystalline parts [225,226]. An overview of the different carbohydrates, alone or in combination, that have been employed as building blocks for the encapsulation of either pure plant bioactive compounds, extracts or essential oils, along with the encapsulation process that was employed and the morphological characteristics of the obtained delivery systems, is given below (Table 2). Emphasis is given to research articles published from 2015 till today.

### 3.4. Polymeric Systems

#### 3.4.1. Polymer-Based Nanoparticles

Polymeric nanoparticles (PNPs) are colloidal solid particles or particulate dispersions with size that ranges from 1 to 1000 nm [258,259,260] that allows them to cross biological barriers [261]. The active compounds can be surface-adsorbed onto the polymeric core or entrapped within, and due to their high biocompatibility and ability to encapsulate compounds of different physicochemical properties, they are considered important carriers for plant ingredients [262]. Furthermore, they can improve the stability of the encapsulated molecules, protect volatile compounds, reduce their degradation rate, and offer slow and controlled release [263]. Polymeric nanoparticles can be generally divided into (a) nanocapsules, in which the active compounds are confined in a cavity surrounded by a polymer membrane and (b) nanospheres, in which the active compounds are evenly distributed in the matrix of the system [259,262,264,265,266,267]. The polymers that are used for the preparation of PNPs can be natural or synthetic. Natural polymers (biopolymers) are isolated from natural sources such as plants, algae, fungi, bacteria and animals, whereas synthetic polymers are often used to modify and improve the structure of natural ones [268]. Moreover, the use of hydrophilic polymers as a matrix for the development of modified-release nanoparticles (MRNs) has also been reported in the literature. The latter provide a controlled and predictable drug release in order to avoid random fluctuations in blood concentration. This can be achieved with the aid of mathematical models that allow the determination of the pharmacokinetics of drugs loaded in nanoparticles toward improving their bioavailability [269]. 

Different methods can be employed to produce polymeric nanoparticles, depending on the type of active ingredient(s) to be loaded and the desired characteristics of the final formulation [270]. The polymerization of monomers and the dispersion of preformed polymers are the two main ways of preparation [271,272]. For nanocapsule preparation, the method of choice is nanoprecipitation whereas for nanosphere preparation, the most commonly used techniques are solvent evaporation, nanoprecipitation, emulsification/reverse salting-out, emulsification/solvent diffusion [273,274]. The first method developed for the formation of polymers was the evaporation of the solvent, where an oil-in-water (*o*/*w*) emulsion is first prepared and then nanospheres are produced [275,276,277]. The polymer is dissolved in a polar solvent and the active ingredient(s) is incorporated by dispersion or dissolution. For application in biomedicine, toxic solvents (chloroform and dichloromethane) have been replaced by less toxic ones (ethyl acetate) [278,279]. With the aid of a surfactant, the organic solution is emulsified in the aqueous phase and then homogenized using ultrasounds or high shear [280]. The solvent gets evaporated at room temperature and the nanoparticles are collected and lyophilized [281]. Another method is emulsification/solvent diffusion where an o/w emulsion is formed between a water phase (with TA surfactant) and a water-miscible solvent that contains the active compounds and the polymer. The internal phase consists of a partially hydro-miscible organic solvent, previously mixed with water at room temperature so that the two phases are thermodynamically balanced [135,282]. Τhe formation of colloidal particles is created from the dispersion of the droplets in the external phase, when a large quantity of water is added and diffusion is caused. The nanospheres generated range in size from 80 to 900 nm. This method is often used despite its great need for water [283,284]. Τhe emulsification/reverse salting-out method creates nanospheres when a hydro-miscible solution is separated from an aqueous one by a salting-out effect. The formation of the o/w emulsion is carried out at room temperature with continuous and intense stirring and then diluted with an aqueous solution which allows the diffusion of the organic solvent, the exterior phase and the precipitation of the polymer. The remaining solvent and the salting-out agent are removed by cross-flow filtration. This method creates nanospheres of size between 170 and 900 nm [285,286]. 

After preparation, characterization of the polymeric nanoparticles is performed mainly by dynamic light scattering (DLS) or photon correlation spectroscopy (PCS), electron microscopy, electrophoresis, near-infrared spectroscopy and chromatography [287,288,289].

#### 3.4.2. Micelles

Polymeric micelles are formed when amphiphilic polymeric molecules bind to an aqueous medium to form vesicles or core-shell structures. Hydrophobic bioactive compounds can be encapsulated in the nucleus and are widely used for passive targeting [290,291]. 10-Hydroxycamptothecin isolated from the plant *Camptotheca acuminata* has been loaded in polymeric micelles and the obtained system demonstrated an inhibitory effect on the activity of glutathione S-transferase with enhanced pharmacokinetics and targeting in liver [292]. The active compound shikonin has been isolated from the plant *Lithospermum erythrorhizon* and loaded into thermosensitive micelles, resulting in its increased biodegradability and solubility as well as its activity against breast cancer cells by temperature regulation [293]. *Sesbania grandiflora* extract has also been encapsulated in polymeric micelles, showing antibacterial activity in an in vitro study against *S. aureus*. The system also offered stability, increased solubility and controlled release [294]. Moreover, an extract of *Posidonia oceanica*, a marine plant rich in carbohydrates and polyphenols that has been shown to exhibit anticancer properties as it inhibits the migration of cancer cells, has been encapsulated in Soluplus polymeric micelles (PM) and chitosan nanoparticles (NP) toward enhancing its bioactivity, aqueous solubility and storage stability [295].

#### 3.4.3. Dendrimers

Dendrimers consist of an inner core and highly branched peripheric structures. Their characteristic is that the active compounds can be incorporated both in the branched surfaces and inside the core by mainly electrostatic or covalent interaction. Their size can be from 1 to 100 nm [291,296]. Dendrimers are characterized by several advantages such as increased solubility, targeting ability, increased half-life, stability, ability to deliver a variety of different active compounds and improved efficiency of delivery [291,297,298,299]. But above all their unique advantages is that they are monodisperse, offering very repeatable pharmacokinetic characteristics [300]. However, the release of the active compounds is ineffective, and the loading of hydrophobic molecules is unstable and poor [301], whereas the production cost remains very high [300]. To address these drawbacks, new categories have been developed such as dendronized polymers or dendrimers incorporating a degradable link [291,296,301]. Curcumin has been incorporated into poly(amidoamine) (PAMAM) dendrimers [302]. An increase in the solubility of curcumin was observed when it was encapsulated in PAMAM, while the system offered controlled release, resulting in a better effect on the antiproliferative activity against lung cancer cells [302,303]. Moreover, in an in vitro study performed on *Plasmodium falciparum*, it was shown that curcumin loaded in dendrimers could be considered an effective anti-*Plasmodium* compound [304]. Silybin, a natural flavonolignan derived from the milk thistle plant, has been encapsulated in PAMAM dendrimers. This resulted in its increased stability, release time and aqueous solubility with a concomitant decrease of the inherent dendrimer cytotoxicity [305]. Black carrot anthocyanins, isolated from *Daucus carota*, have been loaded into silica-PAMAM dendrimers, resulting in the improvement of their solubility and stability as well as their controlled release and cytotoxicity against the neuroblastoma cell line [306]. The bioactive compound liquiritin, isolated from *Glycyrrhiza uralensis*, has been loaded into PAMAM, improving the solubility, stability, biocompatibility and permeability of intestinal absorption [307]. Gallic acid enriched antioxidant dendrimer (GAD) has been used for loading essential oils [308]. Essential oil from the plant *Origanum majorana* has been loaded in PAMAM G4.0 dendrimer and antifungal activity against *Phytophthora infestans* [309]. Moreover, essential oils from the plants *Cymbopogon winterianus* and *Cinnamomum zeylanicum* were encapsulated in four bio-sourced dendrimers. The authors suggested that such delivery systems could find applications in agricultural, food and pharmaceutical industries where the slow release of the active ingredients is required [310].

#### 3.4.4. Polymeric Nanoparticles and Nanogels

Polymeric nanoparticles are colloidal soft particles and their structures can be shell, branched or spherical, with a size ranging from 10 to 100 nm [311]. Nanogels are structures with excellent biocompatibility and targeted bioactive compounds delivery. They are developed in two ways, i.e., the chemical and the physical one [291]. Essential oil derived from the plant *Cymbopogon citratus* has been loaded in poly(d,l-lactide-co-glycolide) nanoparticles and was found to exhibit in vitro anti-herpetic activity and controlled release [312]. 

#### 3.4.5. Nanocapsules

Nanocapsules are nanocolloidal dispersions that have a core-shell structure. The active compounds are encapsulated into a cavity that is externally surrounded by a polymeric coating or polymer membrane. The active compounds may be present in the cavity in aqueous or oily form, in a solid or liquid form. The structure and composition of the core-shell determine its characteristics and the release of the active ingredient it contains.

Depending on the method of preparation, they may be hydrophobic or lipophilic. The main goal is to increase the bioavailability of hydrophilic active ingredients. In addition, they demonstrate high encapsulation efficiency of the active substance due to the increase of solubility of the active compound in the nucleus, low polymer content, protection of polymer shell toward the active substance against degrading agents—such as light, pH—and reduction of tissue irritation due to polymer shell coating [313,314,315]. Extract from the plant *Plumbago europaea* was loaded in poly (lactic acid) (PLA) nanocapsules and antibacterial efficiency was shown: for *E. coli* the efficiency was more than 30% and for *S. aureus* up to 80% [316]. The essential oil from the plant *Achyrocline satureioides* incorporates antioxidant molecules that can be used against oxidative stress, which can cause heart injury during *Trypanosoma evansi* infection. In a study carried out in essential oils encapsulated in nanocapsules, their protective effect against the oxidative stress caused by *T. evansi* was shown [317,318].

#### 3.4.6. Nanospheres

Nanospheres are colloidal aqueous solutions of crystalline or amorphous nature with size from 10 to 200 nm [319]. The main advantage of nanospheres is that they are stable in biological fluids and may improve the bioavailability and control the active compound’s release. Furthermore, nanospheres have reduced toxicity and improvement of entrapment of the bioactive compounds [233]. In an in vivo study in mice, nerolidol, an active ingredient isolated from the ginger plant, was loaded into nanospheres. Its therapeutic efficacy and solubility were improved, and it was able to penetrate the blood–brain barrier [319]. Essential oil from the plant *Zanthoxylum riedelianum* was loaded into nanospheres and the system exhibited improved stability as well as solubility, with controlled release and less photodegradation. The system had insecticidal and insect repellent properties against the species *Bemisia tabaci* [320]. Menthol, an active metabolite isolated from various plants of the Lamiaceae family, was loaded into PLGA nanospheres, gaining enhanced biodegradability as well as controlled release [321].

#### 3.4.7. Nanofibers

Nanofibers are solid polymeric fibers with a small pore size, a large surface area and a size range between 10 and 1000 nm [322]. They have the ability to prevent infection, they have the potential for wound healing, regeneration of damaged tissue and may also demonstrate adhesive features [323,324]. Moreover, anticancer properties of nanofibers upon loading with natural compounds as well as their strong ability to bind to cancer cells have also been shown [225,325,326]. In an in vitro study, nanofibers loaded with an *Aloe vera* extract, intensification of the wound-healing process and repair of the skin were observed, as well as an improvement of the biocompatibility on fibroblast cells [225]. In an in vitro study carried out on nanofibers loaded with an *Lycium barbarum* extract, neuroprotective and peripheral nerve regeneration properties were shown [325]. In another in vitro study, *Cissus quadrangularis* was loaded on nanofibers and an increase of osteogenic differentiation, proliferation and adhesion of mesenchymal stem cells (MSCs) was observed [326].

#### 3.4.8. Polymersomes

Polymersomes are nanospheric vesicles formed by self-assembly of amphipathic block co-polymers. Despite their similarities to liposomes, they are less permeable and more stable. They have the ability to bind to antibodies and to incorporate proteins and non-hydrophilic and hydrophilic bioactive compounds and even DNA and RNA fragments in their membrane [226,327]. Polymersomes loaded with an extract from the plant *Bacopa monniera* demonstrated a significant improvement in memory loss as well as improved targeting of the active compounds in the brain [328]. In another study carried out in mice, curcumin was loaded into polymersomes and showed an affinity for neurons, neuroprotective properties and improved cognitive impairment [329]. An overview of the different polymeric carriers that have been employed for loading either pure bioactive compounds, plant extracts or essential oils is given in Table 3.

### 3.5. Nanoemulsions

Nanoemulsions are colloidal dispersion systems with droplet size up to 100 nm. They are transparent or translucent, optically single isotropic and thermodynamically stable [330]. They can be prepared from aqueous and oily phase and stabilized using surfactants and co-surfactants. They are categorized into oil-in-water (*o*/*w*), water-in-oil (*w*/*o*) and bi-continuous nanoemulsions [331].

Encapsulation of plant isolated ingredients, extracts and essential oils can enhance their stability and effectiveness and make them more effective [332,333,334]. The preparation of nanoemulsions requires a large amount of energy and surfactant as they are non-equilibrated formulations. The technique to prepare nanoemulsions with high energy is the traditional method of making emulsions as well. With high kinetic energy, the size of the microdroplets is reduced to nanodroplets [335]. High-pressure homogenizers, microfluidizers and ultrasounds are used. Other techniques use low energy to prepare nanoemulsions [336]. Low-energy methods are divided into two major categories: those in which the emulsification takes place spontaneously and with an inverse emulsion phase (isothermal) and those that are formed by phase inversion temperature (thermal). The isothermal method does not require a change of temperature or the use of specialized homogenization equipment for the production of fine droplets. The thermal method, on the other hand, requires a change of temperature in order to form a nanoemulsion [337]. Factors influencing the preparation of the nanoemulsions by the low-energy method are the types of surfactants used, the addition of co-surfactant, the ratio of the surfactant to the solvents, the presence of a co-solvent, the type of oil and the conditions of preparation. Nanoemulsions have many advantages, including the improvement of the absorption, dissolution and solubility of the incorporated bioactive ingredients, as well as the potential for prolonged controlled release. They may also facilitate the penetration of bio-membranes and increase the bioavailability of the bioactive compounds that exhibit low solubility, which may be due to the large interfacial area and the nanosize of the droplets. The use of nanoemulsions is generally safe for human health as lipids and oils can be biodegradable, biocompatible and non-mutagenic. Nanoemulsion formulations are also able to reduce the concentration of the bioactive ingredient(s), thus reducing toxicity and offering greater effectiveness [338].

Hydroxysafflor yellow A, isolated from *Carthamus tinctorius*, has been incorporated into a water-in-oil nanoemulsion, showing improved systemic absorption, bioavailability and transport of digested microemulsions [339]. An oil-in-water nanoemulsion loaded with quercetin isolated from nuts and various parts of plants has also been developed. Quercetin was found to be stable with increased skin permeability reaching the systemic circulation [340]. Moreover, in an in vitro study, quercetin was loaded in an oil-in-water nanoemulsion, resulting in enhanced bioavailability in mice when these were tested for anti-obesity efficacy [341]. Quercetin loaded in nanoemulsions has also been examined in other studies, showing enhanced bioavailability, penetration in blood–brain barrier, higher drug release and increased antioxidant activity [342,343,344]. Nanoemulsions loaded with emodin were tested when administered orally and showed enhanced oral bioavailability and transcellular permeation [345]. Nanoemulsions loaded with emodin have been administered orally and showed enhanced bioavailability and transcellular permeation through inhibition of UGT metabolism [345]. Catechin-loaded nanoemulsions have also been administered orally and transdermally and exhibited improved bioavailability, skin permeability and sustained release [346]. In an in vitro study, nanoemulsions loaded with betulinic acid, an antioxidant and hepatoprotective compound, were tested when administered orally. The results revealed enhanced bioavailability, gastrointestinal permeability and sustained release of active compounds [347]. Improved in vivo and in vitro bioavailability and solubility have also been reported for curcumin after its encapsulation in nanoemulsions [348]. β-Elemene showed enhanced in vivo and in vitro antitumor activity against Hep3B cancer cells and solubility after its loading in nanoemulsions [349]. An oil-in-water nanoemulsion loaded with elemene oil obtained from Curcuma species showed improved bioavailability and better stability when administered orally compared to the free form [344]. A nanoemulsion loaded with *O. vulgare* oil has been tested for antimicrobial action in food and appeared to reduce the growth of the bacteria *E. coli*, *S. typhimurium* and *L. monocytogenes* [350]. Moreover, a nanoemulsion loaded with basil oil from the plant *Ocimum basilicum* showed antibacterial activity against *E. coli* [351]. Many types of nanoemulsions have been used for the increase of the physical and storage stability of polyphenols [352]. Curcumin isolated from *C. longa* (turmeric) rhizomes, known for its chemopreventive, anti-inflammatory and anticancer properties [353], has been incorporated into various nanoemulsions [354,355]. In particular, Ma et al. [356] examined several emulsifier types and surfactant-to-oil ratios and assessed the stability of the systems. The authors concluded that the nanoemulsions prepared using Tween-80 as emulsifiers and higher surfactant-to-oil ratios showed improved curcumin storage stability. In a recent study [357], emulsions and nanoemulsions have been used to enhance the chemical stability of curcumin. The authors postulated that droplet size plays the most important role in the degradation of curcumin encapsulated in emulsions; a fact that may affect its bioactivity in various food and beverage products. Resveratrol’s chemical stability may be increased by its incorporation into nanoemulsion compared with that of free (aqueous or ethanolic extract) resveratrol [358]. Phytosterols, such as stigmasterol, β-sitosterol and campesterol, have been proved to inhibit the absorption of dietary cholesterol but demonstrate degradation issues related to oxidation [359]. Their incorporation into nanoemulsions can help overcome this issue. Chuanxun et al. [360] proved the reduction of oxidation degradation of phytosterols caused during storage by using nanoemulsions while Acevedo-Estupiñan et al. [361] prepared phosphatidylcholine and lysophosphatidylcholine nanoemulsions incorporating phytosterols and achieved the increase of their chemical stability as well as their water solubility. Borba et al. [362] prepared β-carotene nanoemulsions by high-pressure homogenization, with an average size of 300 nm [362]. The formulations exhibited increased encapsulation efficiency and stability against droplet coalescence upon storage under different conditions. Qian et al. investigated the influence of temperature, pH, ionic strength, and emulsifier type on the stability of nanoemulsions incorporating β-carotene [363]. Nanoemulsions loaded with D-a-tocopherol (vitamin E) have also been formulated with the aid of high-pressure homogenization [364]. Based on in vitro studies using the Caco-2 cell line in which the prepared nanoemulsions exhibited >90% cell viability, the authors suggested that this system could be used for the delivery of vitamin E after in vivo administration. An overview of the different nanoemulsions that have been employed for loading either pure bioactive compounds, plant extracts or essential oils is given in Table 4.

## 4. Inorganic-Based Delivery Systems

Besides the organic-based delivery systems described in detail above, inorganic materials have also attracted the interest of the scientific community as potential carriers in novel delivery systems for food, pharmaceutical and medicinal applications. However, the use of inorganic delivery systems is still rather limited compared to the organic ones. Some of the most commonly used inorganic carriers are the inorganic nanoparticles, the mesoporous silica nanoparticles (MSNs) as well as the super paramagnetic iron oxide nanoparticles (SPIONs) [376]. Regarding inorganic nanoparticles, they constitute an important class of nanomaterials that due to their small size, high surface area, stability and antimicrobial, antifungal, antivirus and anticancer activity etc., find numerous applications in various fields including food packaging, quality sensing, catalysis, delivery of bioactive compounds etc. [377]. The underlying principle of their synthesis is based on the reduction of the metal ions of a precursor salt solution to zero-valent metal atoms by reducing agents (activation phase). Afterwards, new nanoparticles are formed during the nucleation phase which is followed by the growth phase during which nanoparticles merge to form various morphologies such as spheres, triangles, hexagons, rods, etc. During the last stage (termination phase), the nanoparticles obtain their most stable form with the aid of capping agents (e.g., EDTA, chitosan) [378]. Till recently, inorganic nanoparticles were synthesized via chemical (e.g., electrode position, pyrolysis, microwave assisted combustion) or physical (e.g., colloidal dispersion, vapor condensation) methods. The former involve the use of toxic solvents and reducing agents that are hazardous to the environment whereas the latter involve the use of expensive equipment as well as high temperature and pressure conditions [379]. As an alternative, the biosynthesis of inorganic nanoparticles that is based on the use of biomolecules extracted from plants, bacteria or fungi has attracted the interest of the scientific community during the last decades. Such approaches are simple, take place in aqueous media minimizing the use of unsafe reagents, usually at room temperature or upon mild heating [377]. In particular, plant extracts, containing polyphenols, enzymes, vitamins, etc., have been proved to reduce metal ions as well as to provide stabilization to the formed nanoparticles [380,381]. As Table 5 shows, various nanoparticles composed of silver (Ag-NPs), gold (Au-NPs), palladium (Pd-NPs), zinc oxide (ZnO), silicon dioxide (SiO_2_), titanium dioxide (TiO_2_), etc. have been synthesized using mostly aqueous extracts of different plant materials such as leaves, seeds, fruits, etc. As shown in Table 5, the obtained inorganic NPs may be crystalline or amorphous solids at ambient temperature, may exhibit different shapes (e.g., spherical or non-spherical), surface characteristics and sizes that depend on the raw materials used as well as on the conditions of their fabrication. 

Regarding mesoporous silica nanoparticles (MSNs), they constitute another category of inorganic delivery systems based on their advantageous properties such as controllable morphology, large pore and surface area, biocompatibility as well as ease of surface functionalization. Until now, most of the research studies on MSNs loaded with plant-derived bioactive compounds as delivery systems have been dedicated to cancer therapy [376]. In this regard, spherical mesoporous silica nanoparticles with a size of 60 nm loaded with resveratrol have been fabricated for the treatment of human melanoma [382]. The authors suggested that the encapsulation of resveratrol enhanced its in vitro release properties compared to those of the non-encapsulated molecule whereas in vitro studies revealed that it was found to be cytotoxic against human A375 and MNT-1 melanoma cellular cultures. Resveratrol has also been loaded in uniformly sized (~60 nm) phosphonate and amine modified MSNs in order to improve its in vitro antiproliferative and cytotoxic activity against a prostate cell line. The authors postulated that both phosphonate and amine mesoporous silica nanoparticles showed controlled release compared to the free molecule in 24 h whereas the former were also found to enhance its antiproliferative potential [383]. Mesoporous silica nanoparticles loaded with curcumin have also been fabricated as a potent anticancer agent [384]. Encapsulated curcumin showed increased cellular uptake and cytotoxicity against liver cancer (HepG2) and cervical cancer (HeLa) cell lines compared to free curcumin. Curcumin-loaded MSNs have also been incorporated into chitosan films in order to improve its functional properties toward developing an active food packaging material [385]. It was shown that curcumin loaded in these carriers exhibited pH-dependent and sustained release behavior whereas the prepared films were found to demonstrate antimicrobial activity against *S. aureus* and *E. coli.* MSNs containing eugenol that were prepared by vapor adsorption have also been incorporated into poly(3-hydroxybutyrate-co-3-hydroxyvalerate) (PHBV) films by electrospinning aiming at investigating their potential for active food packaging applications [386]. The electrospun films containing MSNs loaded with eugenol were found to present thermal resistance and enhanced mechanical strength. Moreover, those that contained more than 10% (*w*/*w*) of the MSNs loaded with eugenol were found to inhibit the growth of *S. aureus* and *E. coli* after 15 days. Amino functionalized MSNs have also been prepared as carriers for vitamin E to tackle its poor solubility, instability and low bioavailability [387]. The authors suggested that the encapsulated vitamin E was released in a pH-dependent manner and that after its exposure to air for 48 h it was found to be more stable compared to the free molecule. Apart from pure compounds, MSNs have also been employed for the encapsulation of essentials oils. In particular, Cadena et al. (2019) [388] reported the preparation of such nanoparticles loaded with 41 essential oils from various plant materials including black pepper, ginger, peppermint, garlic, clove bud, rosemary, basil, thyme, sage, mustard, cinnamon, lemongrass, etc. The authors concluded that the encapsulated essential oils exhibited a 10-fold higher antimicrobial activity against *Pectobacterium carotovorum* subsp. *carotovorum* and *Pseudomonas fluorescens* compared to the free ones. 

SPIONs have also attracted the interest of the scientific community due to their small size, biocompatibility and high magnetic moments in the presence of an external magnetic field. Due to these superparamagnetic properties, they find various biomedical applications, e.g., as nano-sensors, cell labeling, tissue repair, as a contrast agent in magnetic resonance imaging, whereas one of their most promising applications is targeted drug delivery based on the magnetic response of the iron oxide, which allows magnetic targeting that makes the retention of nanoparticles in the target tissue longer [389]. The three main iron oxides that have been utilized for the preparation of SPIONs are magnetite (Fe_3_O_4_), maghemite (γ-Fe_2_O_3_) and hematite (α-Fe_2_O_3_). In general, SPIONs can be synthesized via physical (e.g., aerosol, gas phase deposition, pulsed laser ablation), chemical (e.g., co-precipitation, hydrothermal, microemulsion) or biological (e.g., protein, bacteria or fungi mediated) routes. SPIONs loaded with a derivative of trans-resveratrol have been produced using a co-precipitation method [390]. The biological assessment of the efficiency of the synthesized SPIONs was carried out in vitro on C6 rat glioma cells. Results showed that the SPIONs loaded with the derivative of trans-resveratrol did not affect the mitochondrial metabolism using the MTT [3-(4-,5-dimethylthiazol-2-yl)-2,5-diphenyltetrazolium bromide] assay but they were found to damage the plasma membrane using the fluorescein diacetate (FDA) assay at a concentration of 50 µM. The authors suggested that these nanoparticles could have a potential cytotoxic effect that could inhibit the proliferation of cancer cells. Moreover, curcumin-loaded SPIONs have been designed for the examination of the effects of curcumin on testicular hyperthermia in mice that can negatively affect male fertility [391]. In an in vivo study conducted on 18 adult male NMRI mice, protective effects of curcumin-loaded SPIONs on testes damage following hyperthermia have been observed. These effects were attributed to the anti-inflammatory, antioxidant and anti-apoptotic effects of curcumin. Curcumin-loaded SPIONs have also been produced by means of a chemical co-precipitation method and were used for delivery studies against the cervical HeLa cancer cell line. The authors found that the prepared nanoparticles were able to deliver after 6 h, as shown by the increase of the apoptotic cells and of the caspase 3 expression. The preparation of SPIONs loaded with quercetin by means of a nanoprecipitation method has also been reported in the literature [392]. Wistar male rats were orally gavage fed with quercetin, either loaded in SPIONs or in its free form at 50 and 100 mg/kg daily doses for 7 days. A higher concentration of quercetin was observed in the plasma and brain of the rats that were fed with the quercetin-loaded SPIONs compared to those fed with the free molecule. Τhe authors suggested that the use of SPIONs as a targeted drug delivery system enhances the bioavailability of quercetin in the brain ~10-fold higher than the free molecule and could be used for the treatment of neurodegenerative disorders.

In all of the above-mentioned cases, after their synthesis, the obtained inorganic delivery systems are usually characterized by an array of techniques, including UV–Vis spectrophotometry, Fourier-transform infrared spectroscopy (FT-IR), X-ray diffraction (XRD) and scanning electron microscopy (SEM), in order to confirm their successful formation as well as their morphological characteristics (e.g., size, shape, etc.) that determine their unique physicochemical properties and define their gastrointestinal fate and toxicity [393].

## 5. Other Delivery Approaches

Apart from conventional delivery systems categorized into organic and inorganic, contemporary approaches have been developing constantly in order to enhance carriers’ properties. Such approaches are mostly at the stage of fundamental rather than applied research, and currently mostly targeting drug delivery. Nevertheless, it is definite that newer systems will soon begin to have more applications in the delivery of plant ingredients.

In this regard, a different approach to delivery systems constitutes biological nanocarriers, mainly viral nanoparticles (VNPs) and virus-like particles (VLPs). The latter constitute the genome-free versions of their VNP equivalents and are considered non-infectious. The viruses that are used for such purposes are of plant (e.g., tobacco mosaic virus, potato virus X) and mammalian origin or bacteriophages (e.g., MS2, P22). These viruses that range in size (~30 nm up to over 1 µm) currently find applications exclusively in nanomedicine for drug delivery, cancer, antimicrobial, cardiovascular and gene therapies, imaging, vaccines against infectious diseases, etc. The major advantages of such systems are their biodegradability, biocompatibility, water solubility, high loading capacity and uptake efficiency [420]. These facts, along with the relatively easy surface functionalization and the fact that they can encapsulate a broad range of active ingredients, guarantee a promising future for these systems [421].

Another encouraging approach is the advanced drug delivery nanosystems (aDDNSs). These systems consist of the combination of more than one different biomaterials (e.g., lipids, phospholipids, chitosan, dendrimers, etc.) [422]. aDDNSs can be categorized as hybridic and chimeric depending on whether the biomaterials are of the same (e.g., both natural) or different (e.g., one synthetic and one natural) nature [423]. Such mixed systems may offer several advantages. In particular, preclinical studies have shown that aDDNSs can affect the release profile of the entrapped bioactive molecule, alter its pharmacokinetic profile and consequently improve its biodistribution, absorption and metabolism [422].

## 6. Conclusions

There has been increasing interest, during the last decades, in the development of effective delivery systems for plant-derived bioactive ingredients prior to their incorporation into various products in order to overcome some potential challenges related to stability, solubility and bioavailability issues. Organic and inorganic, synthetic and natural, simple and complex and nano- and micro-sized materials have been widely investigated as potential carriers for a broad range of plant ingredients with different physicochemical, biological and functional properties (e.g., colorants, flavoring agents, antioxidants, antimicrobials). In spite of the increasing number of publications related to delivery systems loaded with various plant-derived bioactive compounds, there are still challenges in this field such as the lack of cost-effective methods to scale up production. In this regard, emphasis should be given in the future to the development of large-scale production methods that along with standardization will assist in the commercialization of formulated plant bioactive-loaded delivery systems. In any case, the toxicity of the prepared delivery systems as well as their gastrointestinal fate should be investigated in depth. Moreover, novel approaches (e.g., combinatory and biological systems) are expected to have a key role in the future.

## Figures and Tables

**Table 1:** Overview of different lipid-based delivery systems that have been used for the encapsulation of various pure plant bioactive compounds, extracts and essential oils.

Type of Lipidic Carrier	Encapsulated Material	Target of Encapsulation	Size of the Obtained Delivery System	Application	Reference
Liposome	Quercetin	Solubility	107–139 nm	Oxidative stress and enhanced internalization by cells	[56]
Liposome	Quercetin	Solubility	75–150 nm	Antioxidant activity and stability	[55]
Liposome	Curcumin	Solubility, stability and biocompatibility	350–600 nm	Antioxidant activity and stability	[58]
Liposome	Quercetin	Solubility	50–300 nm	Anticancer and treatment of glioma	[59]
Liposome	Curcumin	Solubility, stability and bioavailability	100–200 nm	Anti-inflammatory activity, sustained-release properties and increased antioxidant activity	[60]
Liposome	Curcumin	Solubility	200 nm	Antioxidant activity and anti-inflammatory	[61]
Liposome	Curcumin	Solubility	182.4 ± 89.2 nm	Anti-inflammatory	[62]
Liposome	Curcumin	Bioavailability	147 ± 6 nm	Wound healing, antibacterial activity and biocompatibility	[63]
Liposome	Curcumin	Solubility and bioavailability	121.81 ± 9.78 nm	Hepatoprotective	[64]
Liposome	Curcumin	Solubility and bioavailability	82.37 ± 2.19–92.42 ± 4.56 nm	Anticancer (skin)	[65]
Liposome	Curcumin	Bioavailability and stability	51.75–140.35 nm	Anticancer (skin)	[66]
Liposome	Curcumin	Bioavailability	>270 nm	Anticancer	[68]
Liposome	Curcumin	Solubility and delivery	420–600 nm	Anticancer (cytotoxicity in lung and colon cancer)	[69]
PEGylated liposomes	Resveratrol	Stability and biocompatibility	86 ± 2.7–171 ± 27.8 nm	Oxidative stress (in vitro and ex vivo)	[46]
Liposome	Resveratrol	Bioavailability and solubility	182.3 ± 12.1–211.2 ± 0.8 nm	Anticancer (brain)	[73]
Liposome	Resveratrol	Solubility and bioavailability	206 ± 10–225 ± 10nm	Antioxidant activity and anti-inflammatory	[74]
Liposome	*O. stamineus* extract	Solubility	152.5 ± 1.1 nm	Antioxidant activity	[78]
Liposome	Green tea polyphenols	Stability, bioavailability and biotransformation	64.5–252 nm	Antioxidant activity and controlled release	[79]
Liposome (soy lecithin liposomes)	Green tea polyphenols (catechin and epigallocatechin gallate)	Stability and shelf- life	- ^a^	Stability	[80]
Liposome	Curcumin	Solubility and bioavailability	45–130 nm	Anticancer (brain)	[71]
Liposome	*P. notoginseng* saponins	Bioavailability, stability and in vitro release	337.8 ± 40.2–117.1 ± 9.7 nm	Edema of brain and reduce the infarct volume	[82]
Liposome	*P. notoginseng* saponins	Bioavailability	40nm	Absorption from intestinal tract in rats	[83]
Liposome	*H. sabdariffa* extract	Stability	46 nm	Higher oxidative stability	[84]
Transfersomes	Caffeine and minoxidil	Stability and release	- ^a^	Alopecia	[96]
Transfersomes	Apigenin	Stability and release	35.41 nm	Skin cancer	[97]
Transfersomes	Epigallocatechin-3-gallate (from *C. sinensis*) and hyaluronic	Solubility and stability	101.2 ± 6.0 nm	Antioxidant and anti-aging properties (antioxidant and anti-aging effects in UV radiation induced skin damage)	[163]
Ethosomes	Caffeic acid	Stability	200 nm	Antioxidant	[99]
Ethosomes	Ginsenoside from *P. ginseng*	Delivery	108.5 to 322.9 nm	Enhanced skin permeation, retention and deposition in vitro	[100]
Νiosomes	Herbal constituents	Solubility, bioavailability, controlled release and stability	- ^a^	Blood–brain barrier targeted delivery	[104]
Νiosomes	*M. communis*	Solubility and permeability	5.3 ± 0.3 to 15.9 ± 2.2 μm	Antimicrobial activity	[105]
Νiosomes	Flavonoid morusin	Solubility and controlled release	400–500 nm (479 nm)	Antimicrobial activity	[106]
Liposomes	Apigenin	Bioavailability	304.10–361.46 nm	Anti-inflammatory	[164]
Nanocrystals	Apigenin	Bioavailability	439 ± 20 nm	Antioxidant activity	[165]
Solid Lipid Nanoparticles	Epigallocatechin-3-gallate (EGCG)	Biocompatibility and toxicity	144–134 nm	Antiproliferative effect	[135]
NLC	Silymarin	Bioavailability, controlled release	213.6 ± 16.0 nm	Used as model	[152]
NLC and SLN	Quercetin	Bioavailability, loading efficiency	67.46–74.61 nm	Brain cancer	[154]
NLC	Curcumin	Cell penetration	100–1250 nm	Breast cancer	[155]
NLC	Curcumin	In vitro digestion, controlled release	225.8 ± 2.3 nm	Used as model	[156]
NLC	Curcumin	In vivo antiplasmodial activity, controlled release	145 nm	Malaria	[157]
NLC	Curcumin and partially hydrolyzed ginsenoside	Bioavailability, controlled release	150–200 nm	Used as model	[158]
NLC	*H. sabdariffa* extract	Bioavailability, encapsulation efficiency, stability	470 ± 8–344 ± 12 nm	Used as model	[159]
NLC	Cinnamon essential oil	Protection and stability	100 ± 1–120 ± 10 nm	Food beverages	[160]
NLC	Peppermint essential oil	Bioavailability, protection	40–250 nm	Antimicrobial, wound healing	[161]
NLC	Sucupira essential oil	Controlled release	148.1 ± 1 nm	Diabetes mellitus	[162]

^a^ Not mentioned.

**Table 2:** Overview of different carbohydrates that have been used as wall materials for the encapsulation of various pure plant bioactive compounds, extracts and essential oils.

Carbohydrate as Wall Material	Carbohydrate Origin and Characteristics	Core Material	Encapsulation Process	Type of the Obtained Delivery System	Morphological Characteristics of the Obtained Delivery System	Application	Reference
Starch	Starch from water chestnut seeds, horse chestnut seeds and lotus stem	Resveratrol	Ultrasonication method	Nanocapsules	419, 797 and 691 nm, increased amorphous character	Controlled released in intestinal juice Anti-obesity and anti-diabetic activity after digestion compared to that of free resveratrol	[227]
Starch	Starch from horse chestnut, water chestnut and lotus stem	Catechin	Ultrasonication	Nanoparticles	322.7, 559.2 and 615.6 nm	Increased bioaccessibility upon in vitro digestion and cell permeability of catechin	[228]
Starch	Starch from pea, corn and potato	Quercetin (standard)	Nanoprecipitation	Nanoparticles	Non-uniformly shaped and nanofiber-like nanoparticles (500 nm) from pea, corn and potato starch, respectively	Increased in vitro antioxidant activity	[229]
Starch	High-amylose corn starch with 70% amylose and low-amylose potato starch	Vitamin D_3_	Ultrasonication	Nanoparticles	32.0–99.2 nm	Increased thermal stability	[230]
Starch	Modified (extruded)	*H. sabdariffa* extract	Spray drying	Microparticles	Oval or round, <10 μm	Antimicrobial activity mainly against L. monocytogenes, E. coli, S. aureus and S. tiphymurium	[231]
Starch	Modified from rice starch	Anthocyanin extract from purple rice bran	Spray drying	Microparticles	Spherical, 6.4 μm	Storage stability of anthocyanins at 4 °C, then at 25 °C, for 90 days Effect on the steady-shear rheology of the rice dough	[232]
Starch	Dafozhi, damaling and daguo starches (amylose contents of 33.5%, 26.7% and 29.8%, respectively)	*G. biloba* extracts	Nanoprecipitation	Nanospheres	Spherical, 255–396 nm	Improved sustained release in artificial gastric and intestinal juices compared to the free extracts	[233]
β-Cyclodextrin	β-Cyclodextrin (purity 98%)	Curcumin	Inclusion complexation	Particles	2–3 µm	Enhanced aqueous solubility Sustained release of curcumin over a period of 5 h	[234]
β-Cyclodextrin	Methylated-β-cyclodextrin, Mw = 1191 Da	Resveratrol	Inclusion complexation	Particles	Irregular shape	Improved solubility Antibacterial activity against *Campylobacter* spp. Preservation of the antioxidant activity	[235]
β-Cyclodextrin with β-glucan	- ^a^	Saffron anthocyanins	Spray drying	Microcapsules	Irregular shape, <124 µm	Release of the maximum amount of anthocyanins during 2 h of simulated intestinal conditions	[236]
Maltodextrin	Maltodextrin	Saffron aqueous extract	Nano-spray drying	Nanoparticles	Spherical, 1.5–4.2 µm	Enhanced stability under in vitro digestion conditions compared to unencapsulated saffron extracts	[237]
Maltodextrin	Commercial maltodextrin, 4-7 DE	Pineapple peel hydroalcoholic extract	Spray drying	Microparticles	Spherical, 18.2 µm	Stable antioxidant activity upon storage for six months at 5 °C	[238]
Chitosan	Low molecular weight chitosan	Curcumin	Ionic gelation	Nanoparticles	Spherical, 167.3–251.5 nm	Enhanced: drug release transdermal permeation and % cell viability of human keratinocyte (HaCat) cells	[239]
Chitosan and pectin	Low molecular weight chitosan from shrimp (deacetylation degree 94.87%) and commercial grade low-methoxy pectin from citrus peel (degree of esterification 2.9%)	Garlic and holy basil essential oils	Ionic gelation	Hydrogel beads	Globular, smooth bead surface, 1.65–2.86 mm	Antimicrobial activity against B. cereus, C. perfringens, E. coli, Pseudomonas fluorescens, L. monocytogenes and S. aureus	[240]
Chitosan and gum Arabic	Deacetylation degree 93%	Curcumin	Polyelectrolyte complexation	Nanoparticles	Spherical and smooth, 250–290 nm	Increased in vitro antioxidant activity (DPPH, FRAP assays) of curcumin Delayed release of curcumin in simulated gastrointestinal conditions	[241]
Chitosan	Medium molecular weight chitosan (deacetylation degree 75–85%)	Cardamom essential oil	Ionic gelation	Nanoparticles	50–100 nm	Non-hemolytic and non-cytotoxic behavior on human corneal epithelial cells and HepG2 cell lines Antimicrobial potential against extended spectrum β lactamase producing *E. coli* and methicillin resistant *S. aureus*	[242]
Chitosan	Medium molecular weight chitosan (deacetylation degree 75–85%)	Lime essential oil	Nanoprecipitation	Nanoparticles	Spherical, 6.1 ± 0.4 nm	Antibacterial activity against the food-borne pathogen *Shigella dysenteriae*	[243]
Chitosan	Medium molecular weight chitosan (deacetylation degree 84.8%)	Peppermint and green tea essential oils	Emulsification-ionic gelation	Nanoparticles	Spherical, 20–60 nm	Increased antioxidant activity by ~2 and 2.4-fold for peppermint and green tea essential oils, respectively Antibacterial activity against *S. aureus* and *E. coli*	[244]
Chitosan	Medium molecular weight chitosan (deacetylation degree 75–85%)	*Mentha piperita* essential oil	Sol-gel method	Nanogel	567.1–575.6 nm	Inhibitory effect on biofilm formation against *S. mutans* on the dental surface and potential use as antibiofilm agent in toothpaste or mouth washing formulations	[245]
Pectin and zein	Citrus peel pectin	Resveratrol	Antisolvent precipitation and electrostatic deposition	Nanoparticles	Spherical, 235 nm	Higher in vitro antioxidant activity compared to free resveratrol Higher antiproliferative activity against human hepatocarcinoma Bel-7402 cells compared to free resveratrol	[246]
Pectin with whey protein concentrate	Citrus low-methoxyl pectin (DE 16–20%)	D-Limonene	Nanocomplex formation	Nanoparticles	Spherical, 100 nm	Protection during processing and storage Controlled release	[247]
Pectin, zein and sodium caseinate	Citrus peel pectin	Eugenol	Nanocomplex formation and nano-spray drying	Nanoparticles	Spherical, 140 nm	Stability upon storage at room temperature for 56 days	[248]
Pectin and egg yolk low density lipoprotein	Citrus peel pectin	Curcumin	Heat-induced nanocomplex formation	Nanogels	Spherical, <60 nm	Increased stability under simulated gastrointestinal conditions Controlled release of curcumin	[249]
Pectin and pea protein isolate	High-methoxyl citrus pectin (DE 90%), beet pectin (DE 62%), low-methoxyl citrus pectin (DE 29%), apple pectin (DE 78%)	Curcumin	Nanocomplex formation	Nanoparticles	Spherical, 559.2 ± 6.2 nm	Protection of curcumin against UV light and thermal degradation Delayed release of curcumin upon in vitro gastrointestinal digestion	[250]
Pectin	Citrus pectin	Citrus peel flavonoids	Ionic gelation	Nanoparticles	Spherical, 271.5 ± 5.3 nm	Controlled release in gastrointestinal fluids Improved antioxidant activity	[251]
Pectin with whey protein concentrate (WPC)	Citrus high-methoxyl pectin (DE 71.1%)	Olive leaf extract	Double-layered emulsification	Nanoemulsions	1443 nm	Slower release rate during 20 days storage at 30 °C	[252]
Pectin with whey protein concentrate	Citrus high-methoxyl pectin (DE 71.1%)	Saffron extract	Double-layered emulsification and spray drying	Nanoparticles	Spherical, 482.3–536.3 nm		[253]
Cellulose	Microcrystalline cellulose	*Origanum vulgare*. essential oil	Ammonium persulfate hydrolysis	Cellulose nanocrystals	1.2–2.9 µm	Antimicrobial activity against *S*. *aureus*, *B*. *subtilis*, *E*. *coli* and *S*. *cerevisiae*	[254]
Cellulose	Bacterial cellulose produced by *Komagataeibacter sucrofermentans*	Cinnamon essential oil	Emulsification	Cellulose nanocrystals	Spherical and rod-like, 350–550 nm	Preparation of solid nanoparticles of biological origin as carriers of cinnamon essential oil that could be mixed directly into the food matrix or as films and coatings	[255]
Cellulose with alginate beads	Cellulose nanocrystals	Thyme essential oil	Emulsification	Cellulose nanocrystals	<200 nm	Antimicrobial effect against *Listeria innocua* via in vitro and in situ tests Reduction of the mesophilic total flora on ground meat, packed under vacuum in combination with gamma irradiation, during storage	[256]
Cellulose	Cellulose nanocrystals extracted from pistachio shells	Peppermint oil	Drop-wise addition of a peppermint oil ethanolic solution in cellulose nanocrystals suspension	Cellulose nanocrystals	Rod-like and spherical, 36.6–55.5 nm	Controlled release upon simulated saliva for 160 min	[257]

^a^ Not mentioned.

**Table 3:** Overview of different polymeric carriers that have been used for the encapsulation of various pure plant bioactive compounds, extracts and essential oils.

Type of Polymeric Carrier	Encapsulated Material	Target of Encapsulation	Size of the Obtained Delivery System	Application	Reference
Micelles	10-Hydroxycamptothecin	Solubility, stability and controlled release	340 nm	Inhibitory effect on the activity of glutathione S-transferase with enhanced pharmaco-kinetic and targeting in liver	[292]
Micelles	Shikonin (from *Lithospermum erythrorhizon*)	Solubility, stability and controlled release	53–98 nm	Targeting to breast cancer cells by temperature regulation	[293]
Micelles	*S. grandiflora* extract	Solubility, stability and controlled release	24.95 ± 0.34 nm	Antibacterial activity in an in vitro study against *S. aureus*.	[294]
Micelles	*P. oceanica* extract	Bioavailability, solubility and stability	252–55.74 nm	Anticancer properties as it inhibits the migration of cancer cells	[295]
Dendrimers (PAMAM)	Curcumin (from *C. longa*)	Solubility and controlled release	*-* ^a^	Better effect on the antiproliferative activity against lung cancer cells	[302]
Dendrimers (PAMAM)	Curcumin	Bioavailability, solubility	~150 nm	*-* ^a^	[303]
Dendrimer G2	Curcumin	Solubility	239 nm	Effective anti-Plasmodium compound—against malaria	[304]
Dendrimers (PAMAM)	Silybin (from milk thistle plant)	Solubility, stability and controlled release	*-* ^a^	Drug solubilization/inherent dendrimer cytotoxicity was reduced	[305]
Dendrimers (PAMAM)	Black carrot anthocyanins (from *D. carota* plant)	Solubility, stability, biocompatibility and controlled release	134.8 nm	Cytotoxicity against neuroblastoma cell line	[306]
Dendrimers (PAMAM)	Liquiritin (from *G. uralensis* plant)	Solubility, stability and biocompatibility	- ^a^	Permeability of intestinal absorption	[307]
Dendrimers (PAMAM)	*O. majorana* essential oil	Solubility, stability and volatility	20–30 nm	Action against the fungus *P. infestans*	[309]
Dendrimers	*C. zeylanicum* and *C. winterianus* essential oil	Controlled release	- ^a^	Biopesticides	[310]
Nanoparticles	*C. citratus*	Controlled release	217.1 ± 19.9 nm	In vitro anti-herpetic activity	[312]
Nanocapsules (PLA)	*P. europaea* extract	Controlled release	271.2 ± 13–1750 ± 305 nm	Antibacterial efficiency	[316]
Nanocapsules	*A. satureioides* essential oil	- ^a^	235.9 nm	Oxidative stress	[317]

^a^ Not mentioned.

**Table 4:** Overview of different nanoemulsions that have been used for the encapsulation of various pure plant bioactive compounds, extracts and essential oils.

Type of Nanoemulsion	Encapsulated Material	Target of Encapsulation	Size of the Obtained Delivery System	Application	Reference
W/O ^a^	Hydroxysafflor yellow A	Bioavailability	53.3 nm	Oral bioavailability	[339]
O/W ^b^	Emodin	Oral bioavailability	116 ± 6.5 nm	Inhibition of UGT metabolism	[345]
W/O ^a^	Catechin	Bioavailability	98.6 ± 1.01 nm	Photoprotection against UVA-induced oxidative stress	[346]
W/O ^a^ and O/W ^b^	Betulinic acid	Bioavailability and solubility	150.3 ± 0.56 nm	Hepatoprotective and in vivo antioxidant efficacy activity	[347]
O/W ^b^	Curcumin	Oral bioavailability	11.2 nm	Enhancement in C_max_	[348]
W/O ^a^	β-Elemene	Solubility	52.68 nm	Antitumor activity	[349]
O/W ^b^	Quercetin	Bioavailability and solubility	19.3 ± 0.17 nm	Contribute to preventing weight gain	[365]
O/W/O	Quercetin	Bioavailability and solubility	180–200 nm	(candidate for the treatment of obesity)	[366]
O/W ^b^	Curcumin and quercetin	Simultaneous drug administration and protection of the encapsulated compounds from degradation	112.33 ± 1.51 nm	Protecting against lipid oxidation (chicken paté)	[367]
O/W ^b^	Curcumin and quercetin	Solubility, high encapsulation efficiency and long-term stability	175.44 nm	Thermal stability, higher bioavailability and consequently drug effectiveness	[368]
O/W ^b^	Quercetin	Poor water solubility and high susceptibility to chemical degradation	207–289 nm	Drug delivery system	[369]
W/O ^a^	Quercetin	Solubility	38.9–266.67 nm	Antioxidant and antibacterial activity	[370]
O/W ^b^	Oregano oil	Solubility	148 nm	Antimicrobial activity in food	[350]
O/W ^b^	*Pterodon emarginatus*	Solubility	125 nm	Larvicidal property against *Aedes aegypti*	[371]
O/W ^b^	*Garcinia mangostana* extract	Bioavailability and solubility	181 nm (167.3–222.0 nm)	- ^c^	[372]
O/W ^b^	*Pimpinella anisum* essential oil	Solubility	440 nm	Antimicrobial activity	[373]
- ^c^	Anthocyanin	Bioavailability and stability	- ^c^	Antimicrobial activity	[374]
- ^c^	2,4,6-triphenylaniline (TPA)	Stability and bioavailability	- ^c^	Therapeutic drug delivery system in diabetes mellitus	[375]

^a^ Water-in-oil emulsion; ^b^ oil-in-water emulsion; ^c^ not mentioned.

**Table 5:** Overview of different inorganic nanoparticles as carriers for pure plant bioactive compounds, extracts and essential oils.

Inorganic Material	Core Material	Shape and Size of the Obtained Delivery System	Application	Reference
Silver	Cavendish banana peels	Spherical, crystalline, 55 nm	Antimicrobial activity against *S. aureus*, *B. subtilis*, *E. coli* and *K. pneumonia*	[394]
Silver	*A. vera*	Octahedral, 5–50 nm	Antimicrobial activity against *S. aureus*, *B. cereus*, *Micrococcus luteus*, *E. coli* and *K. pneumonia*	[395]
Silver	*A. vera*	Crystalline, 70–192 nm	Antibacterial activity against *S. epidermidis* and *P. aeruginosa*	[396]
Silver	Tamarind fruit	Spherical, crystalline, 6–8 nm	Antibacterial activity against *B. cereus*, *S. aureus*, *M. luteus*, *B. subtilis*, *Enterococcus* sp., *P. aeruginosa*, *Salmonella typhi*, *E. coli* and *K.* *pneumonia*	[397]
Silver	Cinnamon	Spherical, 50–70 nm	Antibacterial activity against *S. aureus*, *E. coli*, *B. cereus* and *Pseudomonas* species	[398]
Silver	*A. vera*	Spherical, crystalline, <15 nm	Antibacterial activity against *Kocuria varians* and mercury removal capacity	[399]
Silver	White tea leaves	Spherical, 19.8 nm	Antioxidant activity	[400]
Silver	*Plumbago auriculata*	Spherical, hexagonal, <50 nm	Antimicrobial activity against *S. aureus*, *E. coli*, *Klebsiella pneumoniae* and *Bacillus subtilis*	[401]
Silver	*Citrus limon* peels	Spherical, 59.7 nm	Antibacterial and cytotoxic activity	[402]
Silver	Curcumin	Spherical, polycrystalline, 25–35 nm	Antibacterial activity against *P. aeruginosa*, *E. coli*, *B. subtilis* and *S. aureus*	[403]
Silver	Turmeric extracts	Spherical and quasi-spherical, crystalline, 18 nm	Antimicrobial activity against *E. coli* O157:H7 and *L. monocytogenes*	[404]
Silver	*Mentha piperita*	Spherical, 35 nm	Effect on the neurological enzyme acetylcholinesterase to predict its neurotoxicity	[405]
Silver	*Madhuca latifolia* aqueous extract	Spherical, crystalline, 2–30 nm	Antioxidant and antibacterial activity against *E. coli*, *S. aureus*, *L. monocytogenes*, *S. faecalis*, *S. typhimurium*	[406]
Silver and gold	Quercetin	Crystalline 53 and 27, respectively	Anti-neuroinflammatory activity on BV-2 microglial cells	[407]
Gold	*Plumeria alba* flower	Spherical, 15.6–28 nm	Antibacterial activity against *E. coli*	[408]
Gold	*Hibiscus sabdariffa* leaves	Spherical, crystalline, 10–60 nm	Cytotoxic activity against U87 glioblastoma cells under hyperglycemic condition	[409]
Gold	*Mimosa tenuiflora*	Spherical, 20–200 nm	Cytotoxic activity and catalytic properties	[410]
Gold	Resveratrol	Spherical, crystalline, 14.9–16.1 nm	Anticancer activity against human breast, pancreatic and prostate cancer cells	[411]
Gold	*Hibiscus sabdariffa* flower	Spherical, crystalline, 15–45 nm	Anti-acute myeloid leukemia effect in a leukemic rodent model	[412]
Palladium	*Hippophae rhamnoides* leaves	Spherical, crystalline, 10 nm	Catalytic activity for the Suzuki–Miyaura coupling in water	[413]
Palladium	*Chrysophyllum cainito*	Crystalline, 169.2 nm	Catalytic activity for C–C coupling and reduction reactions	[414]
Titanium dioxide	*Salvadora persica* aqueous ethanolic extract	Crystalline,19.8 nm	Antimicrobial activity against *S. aureus* and *E. coli*	[415]
Zinc oxide	*Passiflora caerulea*	Spherical, 70 nm	Antibacterial activity against microbes that cause urinary tract infections (e.g., *E. coli*, *Enterococcus* sp., *Streptococcus* sp.)	[416]
Zinc oxide	*Cassia fistula* and *Melia azedarach*	Spherical, 3–68 nm	Antimicrobial activity against *S. aureus* and *E. coli*	[417]
Zinc oxide	*Sambucus ebulus*	Spherical, hexagonal, 17 nm	Antibacterial activity against *B. cereus*, *S. aureus* and *E. coli*	[418]
Zinc oxide	*Deverra tortuosa*	- ^a^ 9.3–31.2 nm	In vitro cytotoxic activity against two cancer cell lines, i.e., human colon adenocarcinoma Caco-2 and human lung adenocarcinoma A549	[419]

^a^ Not mentioned.

## Data Availability

Not applicable.
